# Experimental and Theoretical Study of the Kinetics of Dimerization of Ammonia at Low Temperatures

**DOI:** 10.1021/acs.jpca.5c03008

**Published:** 2025-07-01

**Authors:** Lok Hin Desmond Li, Kevin M. Douglas, Ffion Hall, Alice Kirker, Luke Driver, Gregory N. de Boer, Nikil Kapur, Julia H. Lehman, Mark A. Blitz, Dwayne E. Heard

**Affiliations:** † School of Chemistry, 4468 University of Leeds, Leeds LS2 9JT, U.K.; ‡ EPSRC CDT in Fluid Dynamics, School of Computing, University of Leeds, Leeds LS2 9JT, U.K.; § School of Mechanical Engineering, University of Leeds, Leeds LS2 9JT, U.K.; ∥ School of Chemistry, 1724 University of Birmingham, Edgbaston, Birmingham B15 2TT, U.K.; ⊥ National Centre for Atmospheric Science (NCAS), University of Leeds, Leeds LS2 9JT, U.K.

## Abstract

The kinetics of the dimerization of NH_3_ in helium and nitrogen bath gas within the supersonic flow of a Laval nozzle were investigated at very low temperatures. Experimentally, the fraction of the NH_3_ monomer, *f*_monomer_, remaining in the flow at 91 K in N_2_ and at 35 K in He for a total bath gas density [M]∼5 × 10^16^ molecules cm^–3^ was monitored using fluorescence from the electronically excited NH_2_ photofragment formed following NH_3_ photolysis at 213 nm. No dimerization was observed up to [NH_3_] = 1 × 10^15^ molecules cm^–3^ for 160 mm downstream of the 91 K N_2_ nozzle, nor up to [NH_3_] = 5 × 10^14^ molecules cm^–3^ for 150 mm downstream of the 35 K He nozzle. Dimerization was observed at higher [NH_3_], being more pronounced at lower temperatures. For the C_s_ and C_2h_ conformers of the NH_3_ dimer, calculations at the CCSD­(T)/aug-cc-pVTZ level gave a zero-point vibrational-energy corrected binding energy of −7.52 and −7.33 kJ mol^–1^, respectively. Energy-grained master equation calculations based on statistical rate theory using the open-source MESMER package were used to calculate rate coefficients for dimerization, *k*_dimer_, over the temperature range *T* = 25–300 K and [M] = 10^13^–10^22^ molecules cm^–3^ for He and N_2_. *k*_dimer_ displayed a negative *T* dependence and a positive [M] dependence and was found to be sensitive to changes in the low-lying vibrational frequencies of the NH_3_ dimer, for example, the inclusion of a hindered rotor potential for the internal twisting mode, which alters the density of states. Using the axial profiles of *T*, [M], and velocity for the Laval nozzles, the calculated values of *k*_dimer_ were used to calculate *f*_monomer_ in the flow for comparison with the experiment. At higher [NH_3_], when dimerization was observed, the calculations significantly underestimated the degree of dimerization taking place in the flow, with a significant increase in the calculated value of *k*_dimer_ required to match the experiment. The reasons for the discrepancy are discussed, for example, errors in the calculation of the density of states for the NH_3_ dimer and the average energy removed per collision by the bath gas at very low temperatures.

## Graphical abstract

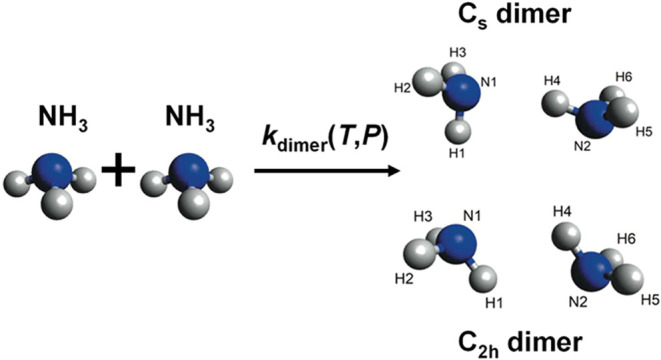

## 1 Introduction

The CRESU (*Cinétique de Réaction de Ecoulement Supersonique Uniforme*) technique is one of the most successful methods for the study of the kinetics of reactions at low temperatures and involves the use of an apparatus with a specially designed convergent-divergent nozzle, known as a Laval nozzle. By flowing from a high-pressure region, known as the reservoir, into the nozzle, the gas begins to accelerate while propagating through the convergent part of the nozzle and expands through the divergent part into a lower-pressure vacuum chamber, forming a cold, supersonic, and uniform flow at the exit of the nozzle.^1–6^ Despite the potentially intensive requirements of the vacuum pumping systems, the Laval nozzle technique has several advantages in the generation of a cold experimental environment over the range ca. 7–180 K. Condensation due to wall effects is absent and the lowest temperature attained is not restricted by the cryogens used, in contrast to cryogenic cooling, and the gas flow is thermally equilibrated in contrast to a free jet expansion where the outcoming flow has significant gradients in both temperature and density.^3–7^

The supersonic flow generated from a Laval nozzle can be used to produce dimers and larger clusters for studies of the nucleation process during phase transitions,^6^ where some examples include water,^8–12^ carbon dioxide,^13–16^ methanol,^17^ and aliphatic^18–20^ and aromatic hydrocarbons.^21,22^ By coupling with mass spectrometry,^11–13,20–22^ X-ray or neutron scattering,^8–10,19^ and Fourier transform infrared spectroscopy,^10,13–19^ properties of the clusters like the size, composition, and structure can be elucidated. The kinetics of the formation of the ethanol–methanol heterodimer has been recently studied by Satterthwaite et al.^23^ with a cryogenic buffer gas cell setup coupled with microwave spectroscopy. The injection of alcohols into the cell was carefully controlled, such that the dimerization reaction was faster than the rate of diffusion. The experimental time scale was lengthened to tens of milliseconds, where the reactants are well thermalized to allow the direct observation of the formation of dimers.

The Laval nozzle is also used to generate a uniform and thermalized cold environment for studies of the kinetics of both ion–molecule and radical–neutral reactions. A detailed list of the reactions studied by different research groups can be found in the review by Cooke and Sims^5^ and in the book by Rowe et al.^6^ Electron beam irradiation can be used for the generation of ions in the gas flow and ion–molecule reactions can be monitored, potentially coupled with ion traps and mass spectrometry.^24,25^ For radical–neutral reactions, laser flash photolysis (LFP) is a common technique to form free radicals from a suitable precursor by directing a pump laser antiparallel or parallel to the gas flow. While other techniques to monitor the reactions include chemiluminescence,^26–29^ mass spectrometry,^30,31^ chirped pulse Fourier transform microwave spectroscopy,^32,33^ cavity ring-down spectroscopy,^34^ and frequency comb spectroscopy,^35^ a popular option coupled with the Laval nozzle to measure the rate coefficient is laser-induced fluorescence (LIF) spectroscopy.^6^ With the use of a second (probe) laser, the fluorescence signals provide a measure of the relative concentrations of the radicals as a function of time (usually a few 100 μs) after the initiation of the reaction, enabling the pseudo-first-order rate coefficient of the radicals for reaction with the added excess coreagent to be obtained. Repeating the experiments with different concentrations of the excess neutral coreagent and plotting the observed pseudo-first-order rate coefficient versus the concentration of the excess coreagent yields a linear bimolecular plot, for which the slope is the measured bimolecular rate coefficient.

It is not uncommon in kinetic studies using Laval nozzles to observe that the bimolecular plots deviate from the expected linear trend, with selective examples including the reactions of CH+CH_2_O,^36^ OH+CH_3_OH,^37,38^ OH+CH_2_O,^39^ OH+CH_3_C­(O)­CH_3_,^40^ and OH+HC­(O)­OCH_3_,^41^ where the slope decreases at higher concentrations of the excess reactant. As is common practice in these and other previous examples,^36,40^ it is essential to disregard the curved portion and only take into account the linear portion at lower excess reactant concentrations, in order to obtain the experimental bimolecular rate coefficient. A plausible explanation for the curvature is that when a higher concentration of excess reactant was used, noncovalently bound dimers or even higher-order oligomers are formed from the monomer of the excess coreagent at the low temperatures of the experiment. Before the establishment of a stable flow, the gas mixture exits the reservoir and expands within the Laval nozzle, where diffusion and potential dimerization simultaneously occur. If the reaction rate coefficient of the dimer (or higher oligomer) with the radical species is not exactly twice that of the monomer (or proportionally larger for oligomers), then the pseudo-first-order rate coefficient will not increase linearly with the excess reagent concentration as further excess reagent is added. In contrast to nucleation experiments within a Laval nozzle where the clusters are deliberately made under higher-pressure conditions,^42^ dimerization of the monomer is a competing and undesirable reaction during kinetic studies of the reaction between the radical and the monomer form of the excess reactant. Avoiding dimerization therefore can restrict the maximum concentration of excess reactants that can be used. In turn, this limits the smallest rate coefficient that can be measured by kinetics experiments within a Laval nozzle, as the time for reaction is already limited by the flow time of the gas along the uniform flow. Therefore, it is important to understand any dimerization of the monomer that may occur and evaluate its impact during kinetic measurements, using both theoretical and experimental approaches, as presented in this work.

The effect of dimerization of methanol (CH_3_OH) in Laval nozzle kinetic experiments has been discussed previously^38,43,44^ in the context of determining the rate coefficient for the OH + CH_3_OH reaction.^37,45^ The noncovalent dimer of methanol has a reported binding energy of ∼18 kJ mol^–1^.^46^ Similar to methanol and ethanol, ammonia (NH_3_) is able to form clusters held together by a network of hydrogen bonds,^47–52^ and there have been several spectroscopic studies of the ammonia dimer.^53–57^ Experiments utilizing infrared excitation coupled with velocity-map ion imaging give a dissociation energy of 660 ± 20 cm^–1^ (7.90 ± 0.24 kJ mol^–1^)^53^ for the ammonia dimer, showing weaker binding than that for methanol. The calculated binding energies (corrected for zero-point vibration) of ammonia dimer from previous theoretical work^58–64^ typically lie in the range of ca. 7–11 kJ mol^–1^.

NH_3_ has previously been used as a reagent in low-temperature Laval kinetics experiments, for example, reactions with CN,^65^ CH,^66^ C_2_H^28^ and OH,^67^ and NH_3_ has been observed both in planetary atmospheres and in the interstellar medium,^68^ and may act as a precursor to prebiotic molecules such as amino acids. NH_3_ has a wide variety of everyday applications, ranging from a feedstock for fertilizers, cleaning agents to pharmaceuticals,^69^ and as a potential zero-carbon fuel, and atmospheric NH_3_ emissions include biogenic, agricultural, and industrial sources.

In the present work, the loss of the NH_3_ monomers in the stable flow region downstream of the exit from a Laval nozzle due to dimerization is monitored via observation of electronically excited NH_2_ which is generated via photolysis of NH_3_. Also, the structure and energetics of ammonia dimers are calculated using ab initio quantum theory, and the rate coefficients for NH_3_ dimerization:

R1 $${\mathrm{NH}}_{3}+{\mathrm{NH}}_{3}+M\left(N_{2}\;\mathrm{or}\;\mathrm{He}\right)\overset{k_{\dim er}\;}{\to }{\left({\mathrm{NH}}_{3}\right)}_{2}+M$$

are calculated as a function of temperature (*T*) and number density of the bath gas M = He and N_2_, [M]. Using the calculated dependence of *k*_dimer_ on *T* and [M], the loss of NH_3_ due to dimerization both within and along the uniform flow exiting the Laval nozzle is calculated, and a comparison of the latter with experiment is presented.

## 2 Methodology

### 2.1 Experimental Studies

Details of the Laval nozzle apparatus used to study the low-temperature kinetics of a range of neutral–neutral reactions have been discussed in detail elsewhere,^36,70,71^ and so only a brief description is given here. Reagent (NH_3_ (99.98% BOC)) and bath gases (either N_2_ (99.9995% BOC) or He (99.9995% BOC)) were combined in a mixing manifold using calibrated mass flow controllers (MKS Instruments) and delivered to a 2 L ballast tank. Following the gas ballast, the gas mixture was pulsed into a 1 cm^3^ stainless steel reservoir and underwent a supersonic expansion through the Laval nozzle into a vacuum chamber, forming a low-temperature jet that extends tens of centimeters downstream of the nozzle exit. Table 1 gives details about the Laval nozzles used in this work. The temperature and density profiles of the flows were characterized by impact pressure measurements, which have been shown to be in good agreement with those obtained by computational fluid dynamics simulations^72^ and rotationally resolved LIF spectroscopy.^36,73^ The pressure in the vacuum chamber, as measured by two calibrated capacitance manometers (Leybold Cerevac CTR100N 0–10 Torr and Leybold Cerevac CTR90 0–1000 Torr), was typically in the range of 0.4–0.9 Torr depending on the nozzle and flow conditions employed and controlled by adjusting the pumping speed on the dry screw pump (Edwards GXS160).

**Table 1:** Properties of the Laval Nozzles Used in This Work

**nozzle name**	**nozzle length** ***L*** **(mm)** ^a^	**bath gas**	**Mach number at nozzle exit**	***T*** **at nozzle exit (K)**	**[M] =** ***n*** **at nozzle exit (molecules cm** ^**–3**^ **)** ^b^
Mach 2.75 nozzle (Leeds)	35	N_2_	3.36	91	4.93 × 10^16^
Mach 4 nozzle (Leeds)	100	He	4.75	35	4.66 × 10^16^
52 K nozzle used by Antiñolo et al.^37^	95.6^c^	N_2_	4.89	52	4.11 × 10^16^

a Defined as the total length from the nozzle entrance to the nozzle exit (see Figure 3).

b From Pitot tube measurements.

c Distance from the sonic point (Mach number = 1) to the nozzle exit

Electronically excited NH_2_ (*A* ^2^A_1_) radicals (hereafter called NH_2_*) were produced from the pulsed laser photolysis of NH_3_ at 213 nm generated by the fifth harmonic of a Nd:YAG laser (Quantel Q smart 850). The photolysis laser had a typical energy of ∼10 mJ per pulse and was introduced colinearly with the axis of the expanded gas flow in order to produce a uniform radical density. NH_2_* fluoresces over a wide spectral range of 290–830 nm in the *A* ^2^A_1_ → *X* ^2^B_1_ system owing to the formation of *A* ^2^A_1_ in many vibrational levels.^74,75^ Fluorescence was collected via a series of lenses and through an optical filter (bandpass interference filter, λ_max_ = 430 nm, fwhm = 10 nm) and detected by a temporally gated channel photomultiplier (CPM; PerkinElmer C1952P) mounted at 90° to the laser beam. The signal from the CPM was recorded using a digital oscilloscope (LeCroy Waverunner LT264) and analyzed using a custom-written LabView program. At each [NH_3_] employed, the fluorescence signal from NH_2_* was averaged for 500 laser pulses.

For a given distance in the uniform flow downstream of the nozzle, the fluorescence signal from NH_2_*, which is proportional to the concentration of the NH_3_ monomer present, is measured as a function of [NH_3_] added in the gas mixture as shown in Figures 1 and S1 (in the Supporting Information, SI) for *z* = 160 mm downstream of the exit of the Mach 2.75 N_2_ nozzle. At smaller [NH_3_] (solid symbols in Figures 1 and S1), the variation is linear, showing there is no loss of NH_3_ owing to dimerization via (R1) as further NH_3_ is added. However, for significantly larger [NH_3_] (open symbols in Figures 1 and S1), there is significant downward curvature in the NH_2_* signal, consistent with the loss of the NH_3_ monomer due to dimerization (R1). Addition of further NH_3_ does not lead to a commensurate increase in the intensity of the NH_2_* fluorescence signal. Any downward curvature due to quenching of NH_2_* by NH_3_ leading to a change in the fluorescence quantum yield was ruled out via observation of the fluorescence lifetime of NH_2_* being constant as [NH_3_] was varied. It is possible that photolysis of the dimer at 213 nm may result in the formation of some NH_2_* fluorescence signal, but the photodissociation quantum yield of NH_2_* from the NH_3_ dimer is likely to be smaller, owing to faster quenching from the larger molecule. It is very unlikely that the photolysis quantum yield of NH_2_* from the dimer is exactly double that from NH_3_, which would lead to a continuation of a linear variation of NH_2_* fluorescence with NH_3_ even if the dimer were being formed. These assumptions are supported by the linear variation followed by the downward curvature of NH_2_* fluorescence with NH_3_ shown in Figure 1. If the equation of the best-fit line in Figure 1 (shown in red) is given by

E1 $$y\left(\mathrm{best}‐\mathrm{fit}\right)=m{\left[{\mathrm{NH}}_{3}\right]}_{\mathrm{added}}$$

**Figure 1:**
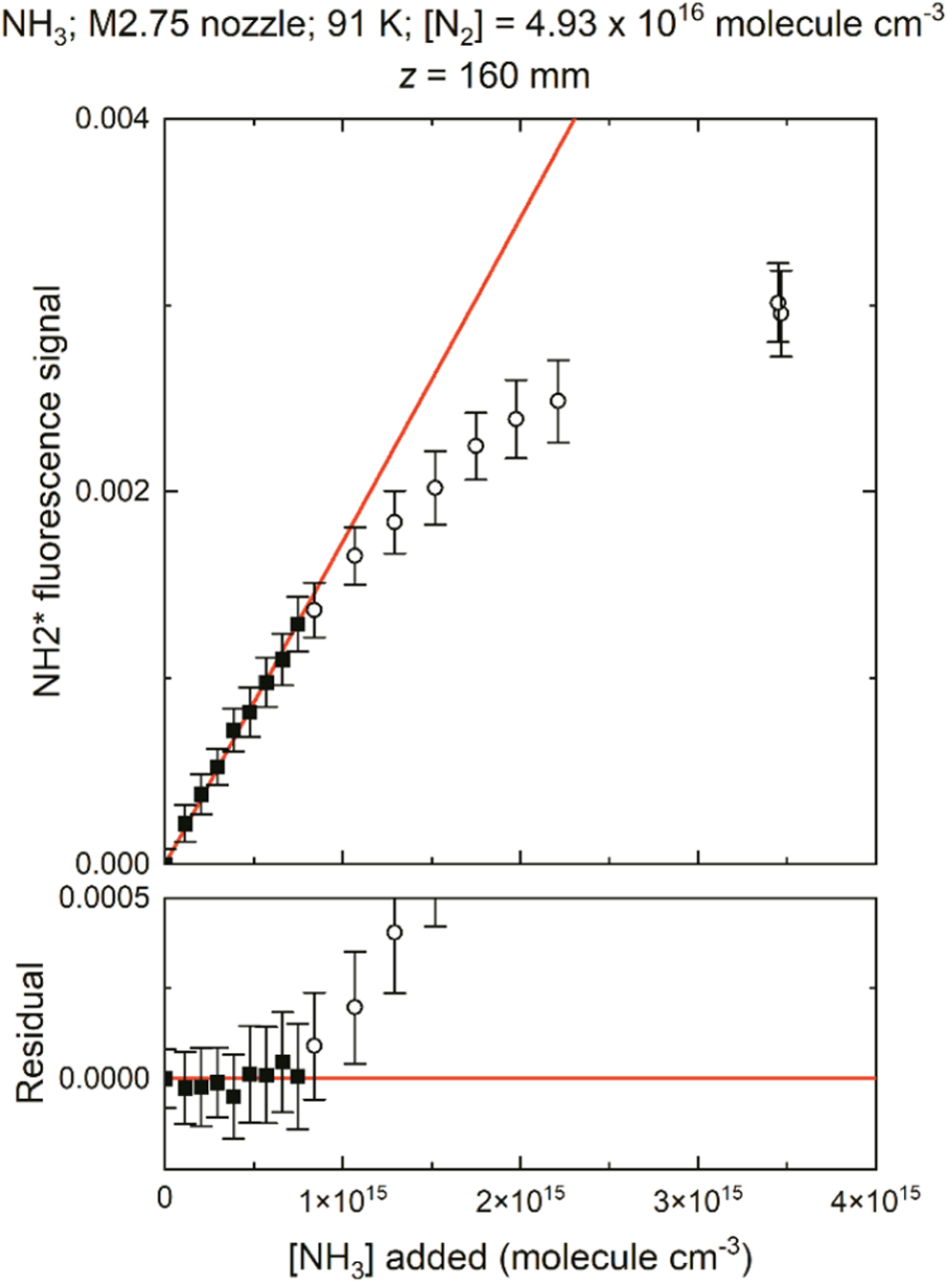
Variation of the NH_2_* fluorescence signal (arbitrary units) at *z* = 160 mm downstream of the nozzle exit versus [NH_3_] added to the flow for the M2.75 nozzle with N_2_ bath gas (top). [NH_3_] is the concentration at the nozzle exit (*z* = 0) calculated from the mole fraction of NH_3_ used to make up the gas mixture and the total density of the flow. At low [NH_3_], the fluorescence signal is linear with [NH_3_] added (closed squares), with a straight line fitted to the closed symbols shown in red. At high [NH_3_], there is downward curvature from the fitted line (open circles). Residuals from the best-fit line (bottom). The error bars represent 1σ error in the fluorescence signal.

then from the experimental results, for each [NH_3_]_added_, we can define the fraction of NH_3_ monomer remaining as

E2 $$f_{\mathrm{monomer}}=\frac{{{\mathrm{NH}}_{2}}^{*}\;\mathrm{fluorescence signal}}{y\left(\mathrm{best}‐\mathrm{fit}\right)}$$

The variation of the NH_2_* fluorescence signal for 3 positions downstream of the nozzle within the uniform flow region of the M4 nozzle with He bath gas is shown in Figure S1 of the SI.

### 2.2 Theoretical Calculations

#### 2.2.1 Electronic Structure Calculations

All ab initio electronic structure calculations were carried out using the Gaussian 09 program.^76^ To determine the effect of different levels of theory used, geometric structures of the monomer and dimer were optimized at the BHandHLYP/aug-cc-pVDZ level^77–80^ and the M06-2X/aug-cc-pVTZ level,^81^ respectively, using the ultrafine integration grids in Gaussian. Using the same level of theory from which the optimized structures were obtained, rotational constants, harmonic vibrational frequencies, and zero-point vibrational energies (ZPVEs) of the respective optimized structures were calculated. The optimized structures were confirmed to be local minima since the harmonic vibrational frequencies were all positive values. ZPVEs obtained from the harmonic frequencies were corrected with a scaling factor of 0.9589 for BHandHLYP/aug-cc-pVDZ^82^ and 0.956 for M06-2X/aug-cc-pVTZ.^83^ Relevant relaxed scans, described below, were also performed at the same level of theory as for the geometry optimization step. More accurate single-point energies, together with the dipole moment values, were obtained at the CCSD­(T)/aug-cc-pVTZ level^84,85^ using the optimized structures.

#### 2.2.2 Rate Coefficient Calculations

Statistical rate theory calculations were subsequently performed using the Master Equation Solver for the Multi-Energy well Reactions (MESMER) program^86^ version 6.1 to obtain the rate coefficient, *k*_dimer_, as a function of temperature and density of the bath gas. The energy-grained master equation was initialized using the ab initio results of the dimerization energies, rotational constants, and harmonic vibrational frequencies which can be included in the form of the Hessian matrix in the input. For the calculation of the density of states, the rigid rotor approximation was applied for all rotations. For the vibrational frequencies of the NH_3_ dimer, apart from those that can nominally be assigned to vibrational motions that already existed within the individual monomers when they are infinitely far apart, there are six additional normal modes which arise due to the interactions between the two monomers. Typically, the additional modes are at lower frequencies due to the weak nature of the intermolecular forces and hence small bond force constants. In particular, one of the motions corresponds to the internal twisting of the two monomers about an axis between them, as shown in Figure 2, which is a hindered rotor motion. Two calculation methods for the density of states were applied to investigate the effect from the different descriptions of the hindered rotor on the rate coefficient. The first method considers all the vibrational modes as harmonic oscillators and the other method describes the new internal twisting mode as a one-dimensional hindered rotor while keeping the rest as harmonic oscillators. Relaxed scans for a full 360° rotation about the dihedral angle corresponding to the twisting motion were performed to obtain the hindered rotor potential to be included in the MESMER input file. With the Hessian matrix provided, MESMER is capable of determining the modes associated with the internal twisting with the projection method reported by Sharma et al.^87^

**Figure 2:**
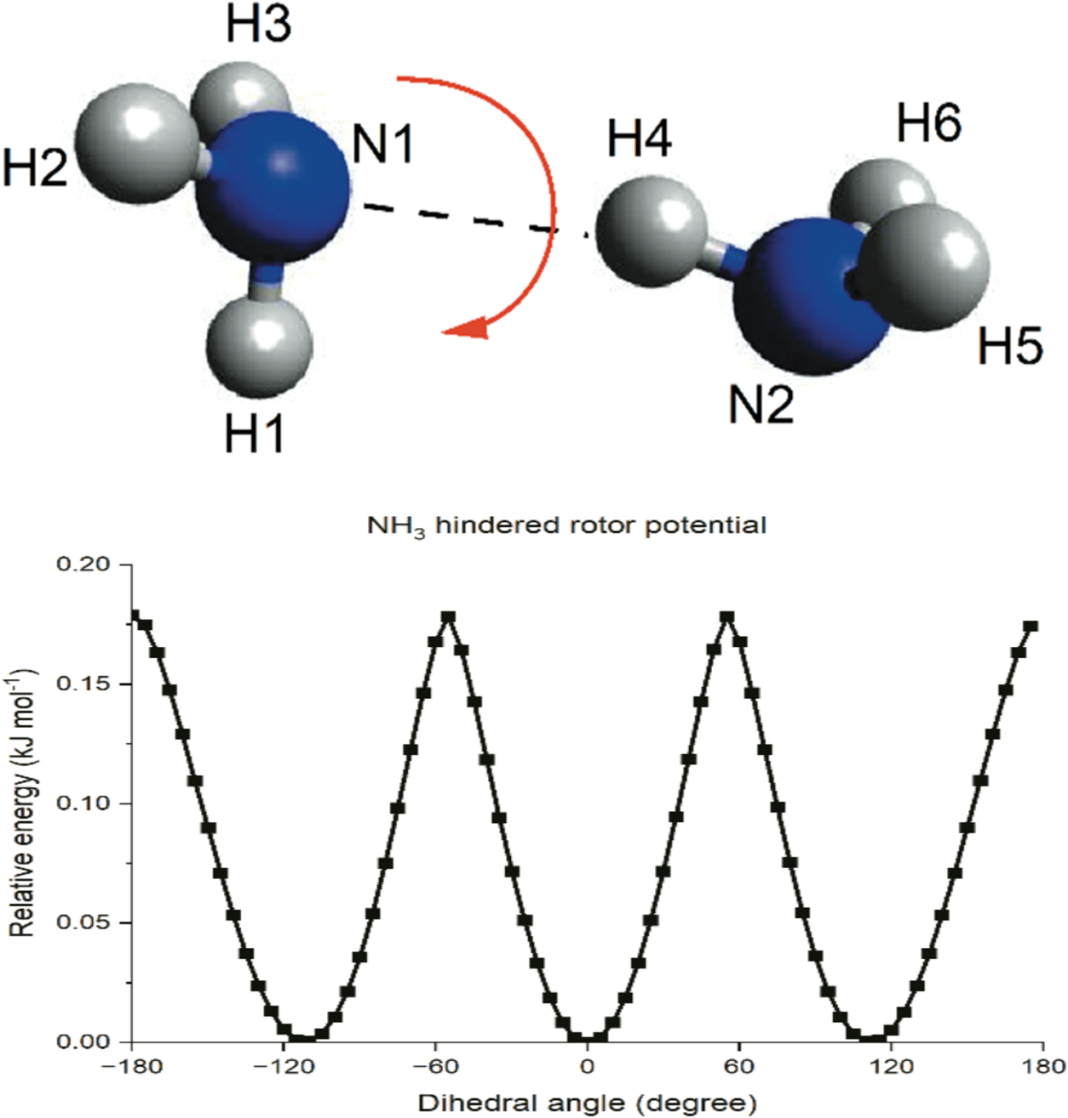
Hindered rotor potential obtained from the relaxed scan about the dihedral angle H1N1H4N2 of the C_s_ dimer at the BHandHLYP/aug-cc-pVDZ level of theory. The optimized equilibrium dimer structure was set at a relative energy of 0 kJ mol^–1^.

As the dimerization reaction is barrierless, the microcanonical rate coefficient of the association process of the two NH_3_ monomers was calculated using the inverse Laplace transformation (ILT) method^88^ from the high-pressure limiting coefficient *k* ^∞^ (*T*), which is taken as the form of a modified Arrhenius expression (with the activation energy being zero):

E3 $$k^{\infty }\left(T\right)=A_{\mathrm{ILT}}^{\infty }{\left(\frac{T}{298K}\right)}^{n_{\mathrm{ILT}}^{\infty }}$$

where *A*_ILT_^∞^ and *n*_ILT_^∞^ are the ILT input parameters for MESMER. In this work, this high-pressure limit was assigned as the collision limit *k*_coll_(*T*) estimated from the classical capture theory.^89–91^ *n*_ILT_^∞^ was assigned to be $-\frac{1}{6}$ , and *A*_ILT_^∞^ could be computed from the dipole moment and mass of NH_3_ (values given in Section 3.2). Further details can be found in SI Section S3, and calculations of *k*_coll_(*T*) are shown in Figure S2.

For the collisional energy transfer process, the probabilities of a given transfer of energy were described using the exponential-down model^92^ with respect to the properties of the bath gases used. In MESMER, the temperature-dependent average energy transferred in each deactivating collision ⟨Δ*E*⟩_*down*_ is modeled as

E4 $${\left\langle \Delta E\right\rangle }_{\mathrm{down}}={\left\langle \Delta E\right\rangle }_{\mathrm{down},\mathrm{ref}}{\left(\frac{T}{298K}\right)}^{n}$$

where ⟨Δ*E*⟩_down,ref_ and *n* are the empirical input values specific to different bath gases listed in Table 2, and further details can be found in the SI Section S4, for example, the Lennard-Jones parameters used for each bath gas in Table S10. The variation of ⟨Δ*E*⟩_down_ with *T* for He and N_2_ bath gases is given in Figure S3. The calculations were performed using a grain size of 30 cm^–1^, which is comparable to the value of the lowest vibrational frequency mode of the dimer.

**Table 2:** Values Used as the Input Parameters in MESMER for the Calculations of ⟨Δ*E*⟩_*down*_ for Different Bath Gases

bath gas	**⟨Δ** ***E*** **⟩**_**down**,**ref**_ (cm^–1^)	***n***
He	100	1
N_2_	250	0.25

#### 2.2.3 Calculations of the Fraction of Monomers in Laval Experiments

Considering a Laval nozzle experiment, after the exit from the reservoir, the reagents have to traverse through two regions: first, the convergent-divergent region within the nozzle, and second, a stable, uniform flow region outside the nozzle where measurements are taken. In the stable region, the flow is collimated and is isentropic. It maintains a constant temperature, number density, and velocity (although some small fluctuations about an average value are present) over some decimeters,^1,2,6,93^ and so these physical properties could be taken as the same as those at the exit of the nozzle (defined here using *z* = 0). However, before the establishment of a stable flow, these physical properties vary inside the nozzle (where *z* is negative) depending on the cross-sectional area of the nozzle along the flow axis.^1,2,93^ In the stable flow region, the concentration of NH_3_ monomer as a function of downstream distance, *z*, depends only on the concentration of NH_3_ exiting the nozzle and any effect of dimerization or diffusional loss out of the uniform flow. However, within the nozzle, the change in NH_3_ concentration due to gas expansion and hence a change in total density, and reactive loss of NH_3_ due to dimerization, both need to be considered. Shannon et al.^38^ evaluated methanol dimer formation using the temperature, number density, and velocity profiles within a 52 K nozzle with N_2_ as the bath gas (details given in Table 1) as provided by the calculations from Antiñolo et al.^37^ (and shown in Figure S8 of the SI), where each of the profiles of these 3 parameters consist of 248 discrete data points, covering a distance of 95.6 mm from the sonic point (at *z* = −95.6 mm, Mach number *M* = 1) to the nozzle exit (defined as *z* = 0), where the Mach number *M* was found to be 4.89. The same calculation method was used in this work to estimate the fraction of ammonia dimers (NH_3_)_2_ as a function of distance *z* that is formed both within the nozzle and in the subsequent uniform flow. At every data point (distance, *z*), we define:

E5 $${\left[{\mathrm{NH}}_{3}\right]}_{\mathrm{original}}=\left[{\mathrm{NH}}_{3}\right]+2\left[{\left({\mathrm{NH}}_{3}\right)}_{2}\right]$$

where [NH_3_]_original_ is the starting NH_3_ monomer concentration at that data point without the dimerization from that data point onward being considered. We also define *k*_dimer_ by

E6 $$-\frac{1}{2}\frac{d\left[{\mathrm{NH}}_{3}\right]}{dt}=\frac{d{\left[{\mathrm{NH}}_{3}\right]}_{2}}{dt}=k_{\dim\mathrm{er}}{\left[{\mathrm{NH}}_{3}\right]}^{2}$$

The fraction of monomer after dimerization for time *t* is defined as *f*_monomer_ = [NH_3_]/[NH_3_]_original_ and from the second-order integrated rate law is given by

E7 $$f_{\mathrm{monomer}}=\frac{1}{1+2k_{\mathrm{dimer}}{\left[{\mathrm{NH}}_{3}\right]}_{\mathrm{original}}t}$$

Assuming no dimerization, in which case *k*_dimer_ = 0, then *f*_monomer_ = 1 and [NH_3_] = [NH_3_]_original_ at all points in the flow. In the uniform flow region, [NH_3_] would be a constant (assuming no significant diffusional loss), but within the Laval nozzle, although [NH_3_] = [NH_3_]_original_, both [NH_3_]_original_ and [NH_3_] are changing with distance owing to expansion, being directly proportional to the total number density.

The fraction of dimer *f*_dimer_ is given by

E8 $$f_{\mathrm{dimer}}=0.5\left(1-f_{\mathrm{monomer}}\right)$$

and so *f*_dimer_ = 0.5 corresponds to the complete conversion of monomers into dimers. As the dimerization in reaction 1 is an association reaction, *k*_dimer_ is a function of both temperature and density [M], and so within the nozzle, the evolving conditions of both temperature and [M] lead to a variable *k*_dimer_. For a small time interval from *t* to *t*+Δ*t*, one can define:

E9 $${\Delta f}_{\mathrm{dimer}}=0.5\left(1-\frac{1}{1+2k_{\mathrm{dimer}}\left(t\right){\left[{\mathrm{NH}}_{3}\right]}_{\mathrm{original}}\left(t\right)\Delta t}\right)$$

*k*_dimer_(*t*) and [NH_3_]_original_(*t*) are functions of time (corresponding to a given distance *z*) and [NH_3_]_original_ (*t*) depends on the conditions within the nozzle as well as the total *f*_dimer_ for times before *t* (*t*′ < *t*) at smaller values of *z*. Given that Δ*t* is sufficiently small, by the best approximation, *f*_dimer_ at a given point (distance, *z*) can be computed as the summation of all Δ*f*_dimer_ from the beginning up to that point. The actual monomer concentration at a given distance in the flow [NH_3_]­(*z*), which is the quantity that is proportional to the fluorescence signal measured in experiments (see Section 2.1), can be calculated for each distance by multiplying *f*_monomer_ by the NH_3_ monomer concentration if there had been no dimerization.

The 52 K nozzle from Antiñolo et al.,^37^ for which a calculated profile of temperature (*T*) and total density is available inside the nozzle, as shown in Figure S8 of the SI, could be used to calculate *k*_dimer_ using MESMER (see Section 2.2.2), and hence calculate *f*_monomer_ at each point inside the nozzle using eq 7. However, the temperature and density profiles inside this nozzle are different from the two nozzles used in the experiments in this work, as given in Table 1. Hence, in order to provide a comparison with experiments, the temperature and [M] profiles inside the 52 K nozzle were scaled such that at the nozzle exit (*z* = 0) the temperature and total density matched the values obtained experimentally in the uniform flow using a Pitot tube (values given in Table 1). Figure 3 shows a schematic of a Laval nozzle, with a distance *z* = 0 corresponding to the nozzle exit, *z* < 0 corresponds to the region within the nozzle itself, while *z* > 0 corresponds to the stable, uniform flow region where experimental studies of the chemical kinetics are performed.

**Figure 3:**
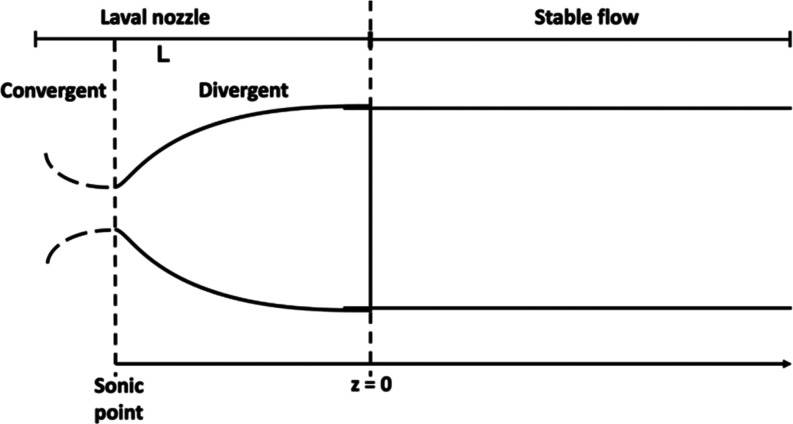
Schematic of the Laval nozzle (total length, L, defined as the distance from the entrance to the exit of the nozzle) to illustrate the definitions of distance along the flow used in this work. The exit of the nozzle is defined as *z* = 0. Within the nozzle, *z* < 0, and in the uniform, stable flow, *z* > 0.

As the Mach number *M*(*z*) at a given distance *z* within the 52 K nozzle has been calculated for each of the 248 data points, the scaling was performed by mapping each (*z*, *M* (*z*)) pair to (*z′*, *M*’ (*z′*)) for the two nozzles where experiments were performed using the following relationships:

E10 $$\frac{z^{\prime }}{-L}=\frac{z_{52\;\mathrm{K nozzle}}}{-95.6}$$

E11 $$\frac{M^{\prime }\left(z^{\prime }\right)-1}{M^{\prime }\left(0\right)-1}=\frac{M\left(z_{52\;\mathrm{K nozzle}}\right)-1}{4.89-1}$$

where information about *L* and *M*’(0) can be found in Table 1. Having calculated a scaled Mach number profile for each of the two experimental nozzles, the temperature and number density profiles can subsequently be constructed by the following formulas that describe the properties of the isentropic core of the Laval nozzle:^1,2^

E12 $$\frac{T_{\mathrm{res}}}{T\left(z^{\prime }\right)}=1+\frac{\gamma -1}{2}{\left(M^{\prime }\left(z^{\prime }\right)\right)}^{2}$$

E13 $$\frac{n_{\mathrm{res}}}{n\left(z^{\prime }\right)}={\left(\frac{T_{\mathrm{res}}}{T\left(z^{\prime }\right)}\right)}^{1/\gamma -1}$$

where γ is the heat capacity ratio of the bath gas. The reservoir temperature *T*_res_ and number density *n*_res_ for a particular nozzle can first be found by substituting *z′* = 0, and then the values obtained can be used to compute *T*(*z′*) and *n*(*z′*) for all the (*z′*, *M*’ (*z′*)) pairs. The gas flow velocity *v* profile can be constructed from the following:

E14 $$\frac{v\left(z^{\prime }\right)}{M^{\prime }\left(z^{\prime }\right)}=\sqrt{\frac{\gamma \mathrm{RT}\left(z^{\prime }\right)}{m}}$$

where *R* is the universal gas constant and *m* is the molar mass of the bath gas used. The scaled profiles of *T*, *n*, *M*, and *v* for the M2.75 nozzle in N_2_ and the M4 nozzle in He are shown in Figures S9 and S10 of the SI, respectively.

As it is not possible to measure the profiles of *T* and total density inside the two nozzles used in the experiments, validation of this scaling approach was performed via a comparison with simulations from computational fluid dynamics (CFD). Previous work has shown very good agreement between experimentally determined and CFD calculated profiles of *T* and total density in the uniform flow region downstream of the Laval nozzle exit,^72^ and hence there is confidence that the profiles calculated by CFD inside the nozzle are also accurate. Details of the CFD calculations are given in the SI Section S8, and Figures S11–S13 show a comparison between CFD calculated profiles of Mach number, *T*, and density with those obtained by scaling the calculated profiles for the 52 K nozzle used by Antiñolo et al.,^37^ with agreement typically within 20%.

Δ*t* in eq 9 is the time taken for the gas to travel from one data point to the subsequent available data point, with the value of Δ*t* chosen to be the time for the gas to flow 1 mm in the stable flow region, and is of the order of 10^–6^ s. Calculations of *f*_dimer_, *f*_monomer_, and [NH_3_] along the flow were performed as a function of the initial ammonia concentration in the flow, and compared with the experimental measurements of these quantities determined using the measured fluorescence from NH_2_*.

The following approximations have been made for the dimerization calculations. First, eq 7 does not account for any dissociation of the collisionally stabilized dimer back to NH_3_ monomers, which is the reverse of reaction 1. Here, Δ*t* is assumed to be small enough that any stabilized dimers remain in dimer form when traveling down the flow. The unimolecular dissociation lifetime of the stabilized dimer was calculated along the flow using MESMER, the details of which can be found in SI Section S10. The lifetime of the dimers was calculated to be considerably longer than the traveling time except for <5 mm downstream of the sonic point within the M2.75 N_2_ nozzle, as shown in Figure S15. Thus, the dimer concentration may be overestimated and the calculated *f*_monomer_ gives a lower limit, particularly at the higher temperatures in the flow closer to the convergent region (Figure 4).^38^

**Figure 4:**
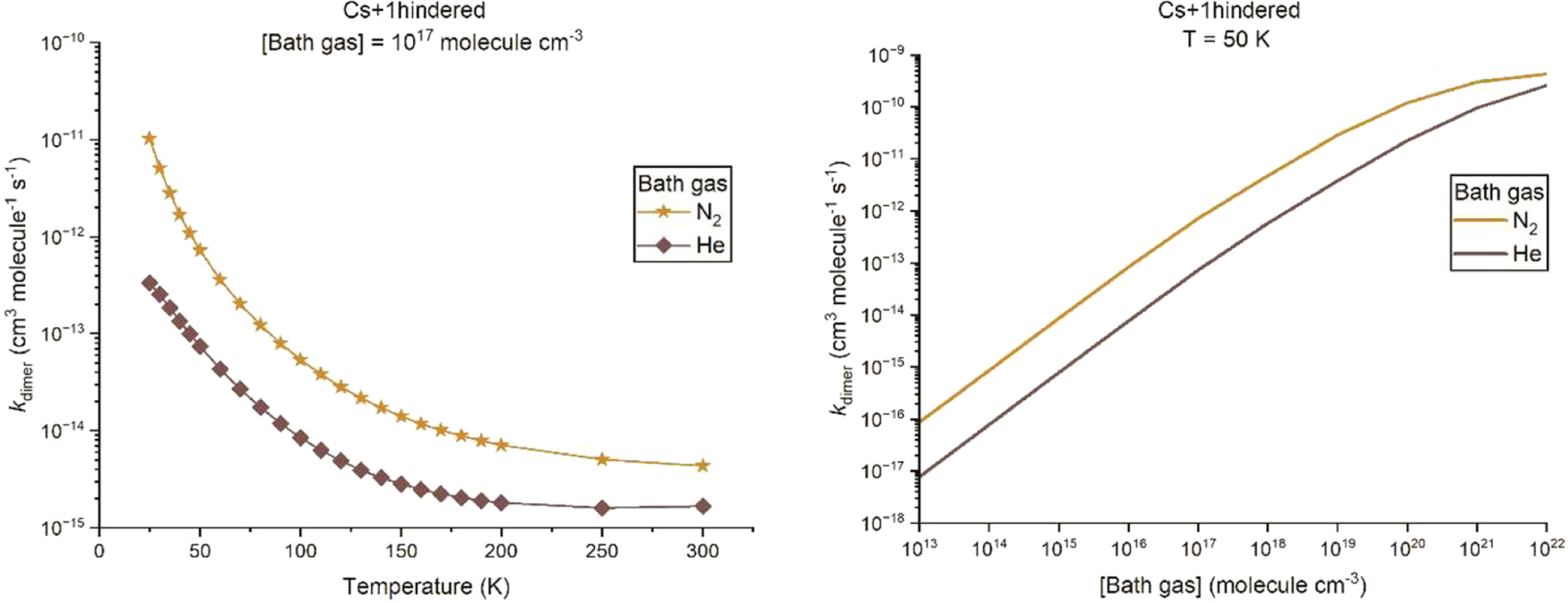
Calculated dimerization rate coefficients, *k*_dimer_, using the “Cs+1hindered” calculation model (see text for details) with N_2_ (amber) and He (brown) as the bath gas, respectively, (left) versus temperature in the range of 25–300 K with a total density of 10^17^ molecules cm^–3^, and (right) versus total number density in the range of 10^13^–10^22^ molecules cm^–3^ with a temperature of 50 K.

Second, the formation of trimers and larger oligomers is neglected, and it is assumed that the formation of dimers is a prerequisite for the formation of further larger oligomers. Finally, any formation of dimers in the convergent region of the flow within the nozzle upstream of the sonic point (see Figure 3) and in the reservoir is not considered in the calculations. This is in part because the calculated temperature and number density profiles for the 52 K nozzle used by Antiñolo et al.^37^ were only performed from the sonic point to the exit of the nozzle, together with the fact that in these regions the temperature is close to room temperature and so the dissociation back to monomers is very fast leading to negligible concentration of the dimer in these regions.

## 3 Results

### 3.1 Electronic Structure Calculations

The optimized structures and geometric parameters of the NH_3_ monomer and dimer calculated at the BHandHLYP/aug-cc-pVDZ and M06-2X/aug-cc-pVTZ levels are illustrated in Table 3, whereas the Cartesian coordinates, vibrational frequencies, and rotational constants can be found in Tables S1–S8 of the SI.

**Table 3:**
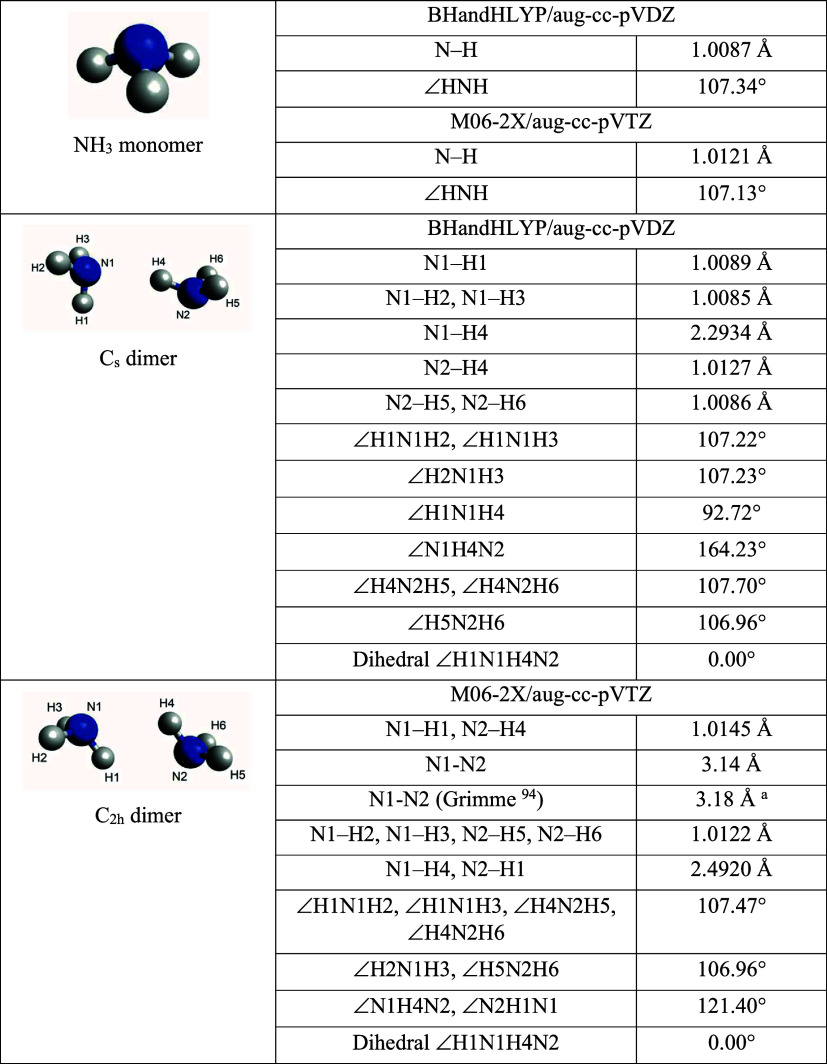
Optimized Structures (Bond Lengths, Angles, Dihedral Angles) of the NH_3_ Monomer and NH_3_ Dimer Obtained at the BHandHLYP/aug-cc-pVDZ and M06-2X/aug-cc-pVTZ Levels of Theory

a Calculated by Grimme^94^ at the DFT-D-PBE level, which applies empirical corrections for the effect of long-range dispersion corrections.

Results from a theoretical study by Tao and Klemperer^58^ provided two possible dimer structures, with C_s_ and C_2h_ symmetry, respectively. Their study suggested that whether one or the other structure is energetically stable with respect to the monomers or which particular structure is the global minimum depends on the level of theory used. Subsequent studies^59–63^ have reported either or both geometries as stable dimer structures, as listed in Table 4.

**Table 4:** Relative Energies (Compared to Two Separate NH_3_ Molecules) (kJ mol^–1^) of the NH_3_ Dimer^a^

	**uncorrected electronic energy of the NH**_**3**_**dimer relative to NH**_**3**_**(corrected value for zero-point energy in brackets)** (kJ mol^–1^)	
**method**	**C_s_**	**C_2h_**
this work 1		–13.47 (−7.33)
this work 2		–13.32 (−7.18)
this work 3	–13.62 (−7.52)	
this work 4	–12.76 (−6.66)	
Tao et al.^58^	–11.52	–11.39
Lee et al.^59^	–13.20	
Kulkarni et al.^60^	–16.86 (−10.88)	–16.02 (−10.59)
Altmann et al.^61^ 1	–13.83 (−7.98)	
Altmann et al.^61^ 2	–15.63 (−9.89)	
Malloum et al.^62^	–13.18	
Yang et al.^63^	–13.63	–13.53

a This work 1: CCSD­(T)/aug-cc-pVTZ//M06–2X/aug-cc-pVTZ. This work 2: M06–2X/aug-cc-pVTZ. This work 3: CCSD­(T)/aug-cc-pVTZ//BHandHLYP/aug-cc-pVDZ. This work 4: BHandHLYP/aug-cc-pVDZ:^58^ MP2/6–311+G­(3d,2p),:^59^ MP2/CBS,:^60^ MP2/6–311++G­(d,p)^61^ 1: MP2/aug-cc-pVTZ,^61^ 2: B3LYP/aug-cc-pVTZ,:^62^ CCSD­(T)/CBS:^63^ Frozen-core UCCSD­(T)-F12a/aug-cc-pVTZ.

It is noted that for the same level of theory, the energy difference between the C_s_ and C_2h_ structures is less than 1 kJ mol^–1^, which is within the computational error, which led Malloum et al.^64^ to suggest that these two structures can be considered as degenerate. This is reasonable due to the similarity of the two geometric structures and the “floppy” nature of the dimer.^95,96^ In this work, when the optimization calculations were performed at the BHandHLYP/aug-cc-pVDZ level, only the C_s_ structure was predicted to be a stable structure, while at the M06-2X/aug-cc-pVTZ level, only the C_2h_ structure was found to be stable. The refined electronic binding energy at the CCSD­(T)/aug-cc-pVTZ level on the two optimized structures gave values of 13.62 and 13.47 kJ mol^–1^, which match with previously reported calculated values, and the ZPVE corrected energy gave values of 7.52 and 7.33 kJ mol^–1^ respectively, close to the reported dissociation energy value of 7.90 ± 0.24 kJ mol^–1^ from experiment.^53^

For the C_s_ structure, the internal twisting motion was found to be along the N - - - H bond, and so a relaxed scan about the H–N - - - H–N dihedral angle was performed, and the resulting hindered rotor potential is presented in Figure 2. The small change in energy (∼0.18 kJ mol^–1^) between the peak and trough of the potential suggests that it is facile for the rotation along the N- - H bond to occur. For the C_2h_ structure, the two N–H bonds (and N- - - H bonds) in the center form a plane at the equilibrium configuration and the internal twisting is about the axis bisecting the two N–H bonds such that they become out of plane. Such rotation breaks the two N- - - H bonds that hold the dimer together. Therefore, only the internal twisting about the H–N - - - H–N dihedral angle for the C_s_ structure will be considered.

### 3.2 Calculations of the Rate Coefficient for NH_3_ Dimerization, *k*_dimer_

The scaled harmonic vibrational frequencies and rotational constants at the BHandHLYP/aug-cc-pVDZ and M06-2X/aug-cc-pVTZ levels, together with the ZPVE corrected energy at the CCSD­(T)/aug-cc-pVTZ level, were used as input for rate coefficient calculations. The dipole moment of NH_3_ at the CCSD­(T)/aug-cc-pVTZ level was found to be 1.5836 and 1.5936 D, respectively, for the optimized structures at the BHandHLYP/aug-cc-pVDZ and M06-2X/aug-cc-pVTZ levels (only ca. 7–8% larger than the experimental value of 1.4758 ± 0.0029 D obtained from Stark spectroscopy),^97^ which then led to values of 6.245 × 10^–10^ and 6.297 × 10^–10^ cm^3^ molecule^–1^ s^–1^ for the ILT input parameter *A*_ILT_^∞^, calculated using eq 3.

The rate coefficients for dimerization, *k*_dimer_, (defined in Section 2.2.3) leading to loss of NH_3_ monomers, were computed in the temperature range of 25–300 K over the number density range of 10^13^–10^22^ molecules cm^–3^ for He and N_2_, which covers the density in the uniform flow downstream of the Laval nozzle (ca. 10^16^–10^17^ molecules cm^–3^), standard atmospheric conditions (10^19^ molecules cm^–3^), and up to even higher pressure where *k*_dimer_ may approach the collision limit. Three different models to calculate the rovibronic density of states were considered. The “C2h” model considers only the C_2h_ dimer optimized at the M06-2X/aug-cc-pVTZ level with the harmonic oscillator approximation applied to all of the vibrational modes. The “Cs” model considers only the C_s_ dimer optimized at the BHandHLYP/aug-cc-pVDZ level with the harmonic oscillator approximation applied to all of the vibrational modes. Finally, the “Cs+1hindered” model differs from “Cs” only in the inclusion of the one-dimensional hindered rotor to describe the internal twisting mode.

The left panel of Figure 4 shows the variation of *k*_dimer_ with temperature for a number density of He or Ar bath gas of 10^17^ molecules cm^–3^, while the right panel shows the pressure-dependent rate coefficients at a fixed temperature of 50 K for He and N_2_ bath gas, both obtained from the “Cs+1hindered” model. The results from all three models used to calculate the density of states and for other pressures can be found in the SI (Figures S4–S6). In general, *k*_dimer_ has a negative temperature dependence over the temperature and number density range studied, as expected for an association reaction proceeding without a barrier. A positive dependence upon the number density is also observed, indicating that the reaction is in the low-pressure regime. As dimerization is a single-step barrierless association reaction, at low temperature and high pressure (number density), stabilization into the potential well of the dimer is favored. The value of *k*_dimer_ in N_2_ bath gas is larger than in He bath gas, which is consistent with their relative strengths as a collider reflected by the ⟨Δ*E*⟩_down_ values as shown in Table 2.

Considering the three different calculation models of the rovibronic density of states, *k*_dimer_ obtained from the “Cs” model is about a factor of 2–4 larger than that from the “C2h” model, and for the “Cs+1hindered” model, *k*_dimer_ is another factor of 2–4 larger. The well-depths for the “C2h” model and the “Cs” model are similar, as indicated by the CCSD­(T)/aug-cc-pVTZ refined energy values, as shown in Table 4. The major difference in the input between the two models lies in the six additional intermolecular normal modes as two different dimer geometries are considered, with the lowest vibrational frequency value being the most noteworthy one. The lowest harmonic vibrational frequency value of the dimer in the “Cs” model (43.6671 cm^–1^) is only ∼50% of that in the “C2h” model (80.7821 cm^–1^), which leads to an increase in the density of states available for the dimer as shown in Figure S7 and contributes mainly to the rise in the dimerization rate coefficient. The inclusion of the hindered rotor potential for the “Cs+1hindered” model further promotes the available density of states and the dimerization rate coefficient, which demonstrates the importance of a more detailed description of the low-frequency modes in avoiding the underrepresentation of the density of states. At high pressure, *k*_dimer_ obtained from the three different models appear to converge as it is approaching the collision limit.

### 3.3 Comparison of Experimentally Measured and Calculated Loss of NH_3_ Monomers

The temperature, number density, and velocity profiles of the 52 K nozzle used by Antiñolo et al.,^37^ together with the scaled profiles for the nozzles used in this work for experiments, can be found in Figures S8–S10. Based on the scaled profiles, *k*_dimer_ along the gas flow within the M2.75 nozzle in N_2_ and M4 nozzle in He was calculated using MESMER for every (*T*, *n*) at each discrete point, and the results are plotted in Figure 5 for the “Cs+1 hindered” model. As the NH_3_ molecules travel along the flow, the two competing factors are a decreasing temperature which favors dimer formation owing to a larger *k*_dimer_ (left panel of Figure 4), and a decreasing total number density due to the expansion within the nozzle which disfavors dimer formation as *k*_dimer_ is approaching the low-pressure limit (right panel of Figure 4). The calculated values of *k*_dimer_ initially decrease downstream from the sonic point within the nozzle (where the number density effect upon *k*_dimer_ outweighs that of temperature) and then increase gradually (where the effect of decreasing temperature dominates) to reach a plateau before reaching the nozzle exit (*z* = 0). After the nozzle exit within the uniform flow, the value of *k*_dimer_ is constant owing to a constant *T* and *n*. A comparison of the calculated profiles of *k*_dimer_ with *z* for both nozzles using the “Cs”, “Cs+1hindered” and “C2h” models is shown in Figure S14 of the SI. The largest value of *k*_dimer_ is obtained using the “Cs+1hindered” model as this calculates the largest density of states of the dimer.

**Figure 5:**
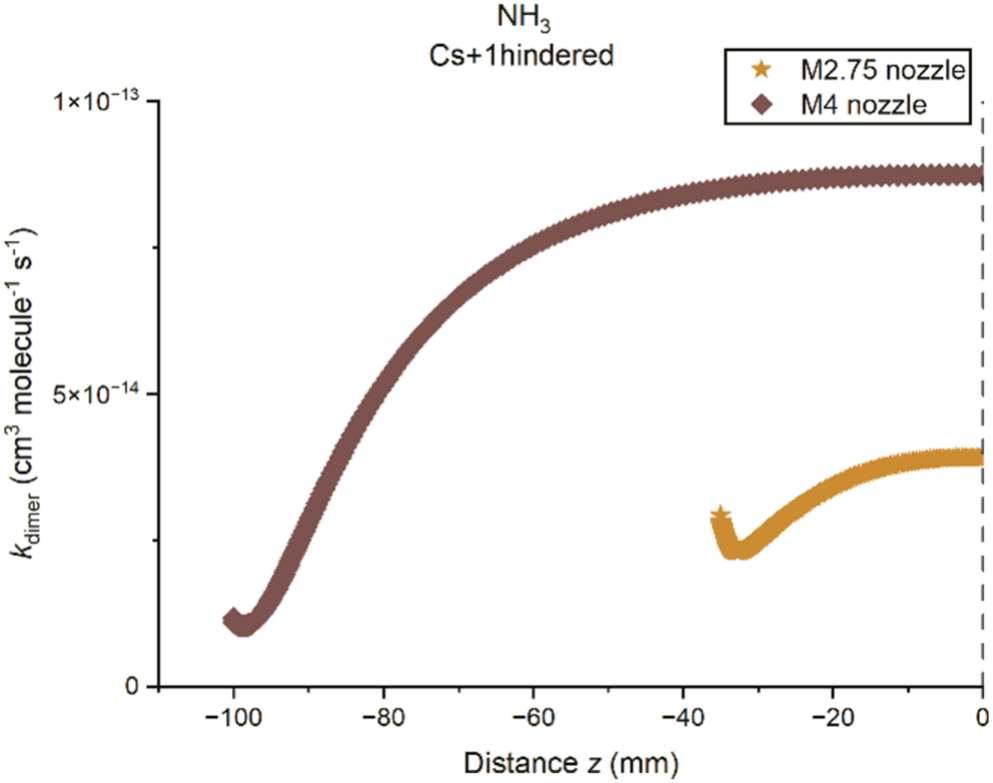
Calculated dimerization rate coefficient *k*_dimer_ in (amber) the M2.75 nozzle in N_2_ (*T* = 91 K, density = 4.93 × 10^16^ molecules cm^–3^ in the uniform flow) and (brown) the M4 nozzle in He (*T* = 35 K, density = 4.66 × 10^16^ molecules cm^–3^ in the uniform flow) as a function of flow distance *z* (*z* = 0 at the nozzle exit) using the “Cs+1hindered” calculation model (see text for details). The M4 nozzle is considerably longer than the M2.75 nozzle.

Based on the values of *k*_dimer_ obtained from Figure 5, *f*_dimer_ was computed using eq 8 in Section 2.2.3 as a function of distance *z* for different concentrations of NH_3_ used. The calculations covered the region from the sonic point within the nozzle to the exit of nozzle and then downstream in the uniform, stable flow region after the nozzle, where it is assumed that the temperature and density, and therefore the value of *k*_dimer_, are constant. As demonstrated in Figure 6, which shows the results from the “Cs+1hindered” model (and Figures S16 and S17 which show the results from the “Cs” and “C2h” models), *f*_dimer_ increases slowly and steadily all the way along the flow when a low [NH_3_] is used, while for higher [NH_3_], a more significant initial rise can be observed before a more gradual increase.

**Figure 6:**
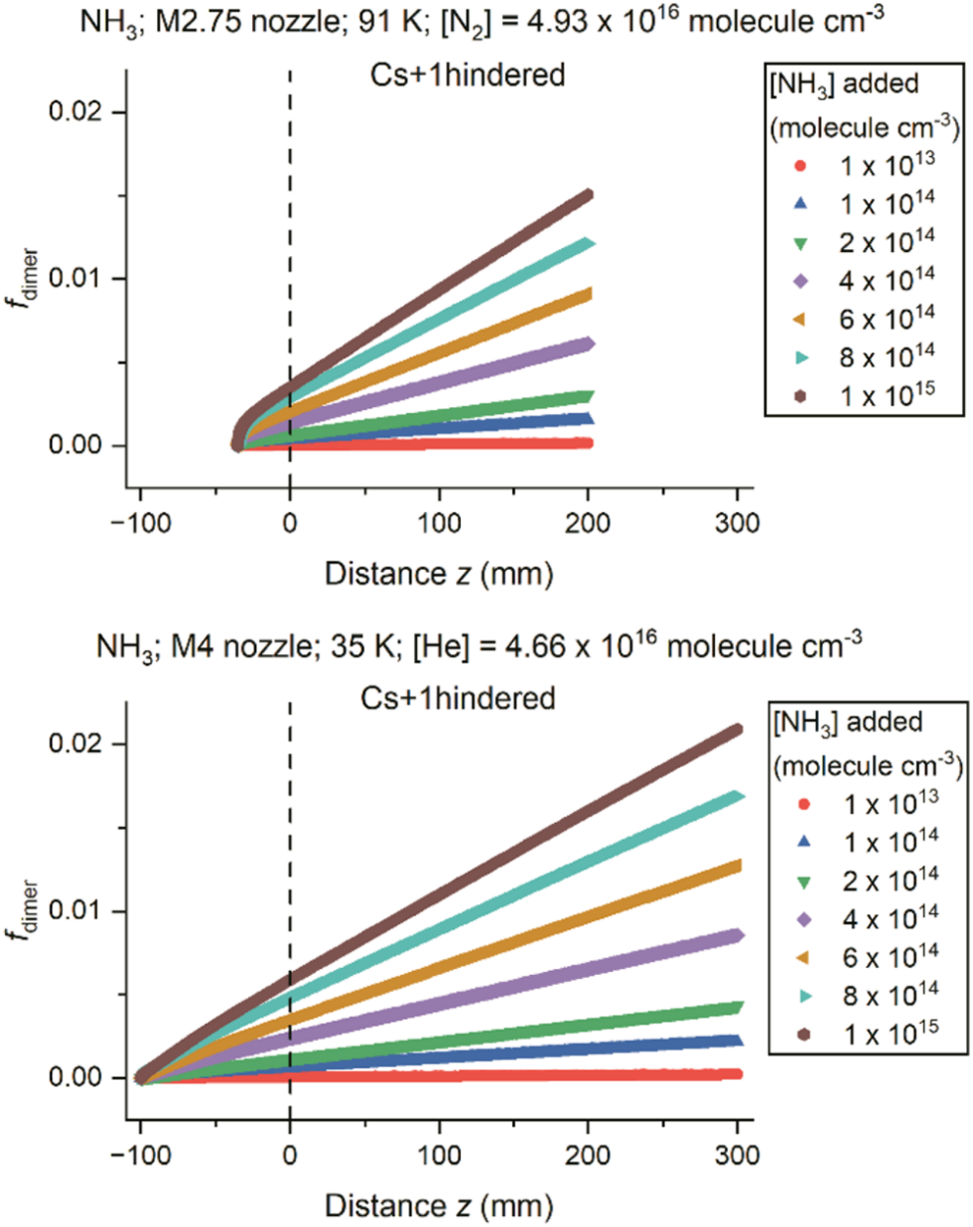
Calculated fraction of NH_3_ dimers, *f*_dimer_, along the gas flow as a function of distance *z* with different monomer concentrations using the “Cs+1hindered” model for (top) the M2.75 nozzle in N_2_ (*T* = 91 K, density = 4.93 × 10^16^ molecules cm^–3^ in the uniform flow) and (bottom) the M4 nozzle in He (*T* = 35 K, density = 4.66 × 10^16^ molecules cm^–3^ in the uniform flow). The NH_3_ monomer concentrations in the legend correspond to the added concentration of NH_3_ (in molecules cm^–3^) at the nozzle exit if there had been no dimerization. The vertical dashed line at *z* = 0 indicates the nozzle exit.

The variation of *f*_monomer_ against distance *z* for different concentrations of NH_3_ used using the three different calculation models (“C2h”, “Cs”, and “Cs+1hindered”) can be found in the SI (Figures S18–S19). *f*_monomer_ for the two nozzles used at selected distances *z* obtained from the “Cs+1hindered” model are shown in Table 5. Considering an added concentration of [NH_3_] = 1 × 10^15^ molecules cm^–3^ at the nozzle exit, which is approximately 2% of the flow and the maximum of typical concentrations of reagents used in chemical kinetics studies using the Laval nozzles, for the “Cs+1hindered” model, *f*_monomer_ is calculated to be 99.30% for the M2.75 nozzle in N_2_ and 98.82% for the M4 nozzle in He, respectively, at the nozzle exit (*z* = 0). At *z* = 50 mm, *f*_monomer_ drops to 98.71 and 98.31%, respectively, for these two nozzles, and at *z* = 100 mm, it further decreases to 98.13 and 97.80%, respectively. A comparison of dimer loss within the nozzle and in the uniform flow is given in Table S11 of the SI.

**Table 5:** Calculated Fraction of NH_3_ Monomers *f*_monomer_ (in %) at Selected Distances *z* with Different Monomer Concentrations Using the “Cs+1hindered” Model for the M2.75, *T* = 91 K Nozzle in N_2_ and the M4 Nozzle in He at *T* = 35 K

M2.75 nozzle (*L* = 35 mm, *T* = 91 K, [N_2_] = 4.93 × 10^16^ molecules cm^–3^)						
	at *z* (mm)					
[NH_3_] added (molecules cm^–3^)	–35	0 (nozzle exit)	50	100	150	200
10^13^	100	99.993	99.987	99.981	99.975	99.969
10^14^	100	99.929	99.870	99.810	99.750	99.691
10^15^	100	99.297	98.710	98.130	97.556	96.990
10^16^	100	93.187	88.261	83.830	79.823	76.182

M4 nozzle (*L* = 100 mm, *T* = 35 K, [He] = 4.66 × 10^16^ molecules cm^–3^)								
	at *z* (mm)							
[NH_3_] added (molecules cm^–3^)	–100	0 (nozzle exit)	50	100	150	200	250	300
10^13^	100	99.988	99.983	99.978	99.972	99.967	99.962	99.957
10^14^	100	99.882	99.829	99.776	99.724	99.671	99.619	99.566
10^15^	100	98.823	98.309	97.801	97.298	96.800	96.307	95.819
10^16^	100	88.839	84.853	81.209	77.866	74.787	71.942	69.306

These results show that dimers are formed both within the nozzle itself and downstream of the nozzle exit in the uniform flow region. For the same distance traversed, more dimers are formed within the nozzle than in the uniform flow region, as shown in Table S11 and Figures S18–S19. Only a very small percentage of dimers form both within the nozzle and the uniform flow, even at the higher end of NH_3_ concentrations used in kinetics studies, consistent with findings of Shannon et al.^38^ for the formation of CH_3_OH dimers.

The left panel of Figure 7 shows the fraction of NH_3_ that remains as the monomer *f*_monomer_ at *z* = 160 mm as a function of the [NH_3_] added for the M2.75 nozzle in N_2_. The experimental values of *f*_monomer_ are from equation ([Disp-formula eq2]) which uses the NH_2_* fluorescence signal as a function of added [NH_3_] from Figure 1. The theoretical values of *f*_monomer_ are calculated from equation ([Disp-formula eq7]) using the “Cs+1hindered” model. The left column of Figure 8 shows the experimental and theoretical values of *f*_monomer_ for the M4 nozzle in He at *z* = 50, 150, and 250 mm (experimental data available for 3 distances). The corresponding results for the “Cs” and “C2h” theoretical models can be found in Figures S20 and S21 of the SI. The NH_2_* fluorescence signal and the calculated [NH_3_] (“Actual”, obtained from multiplying *f*_monomer_ for the given value of *z* by [NH_3_]­(*z*) assuming there had been no dimerization) versus the [NH_3_] added are shown in the right panel of Figure 7 for the M2.75 nozzle in N_2_ at *z* = 160 mm and the right column of Figure 8 for the M4 nozzle in He at *z* = 50, 150, and 250 mm.

**Figure 7:**
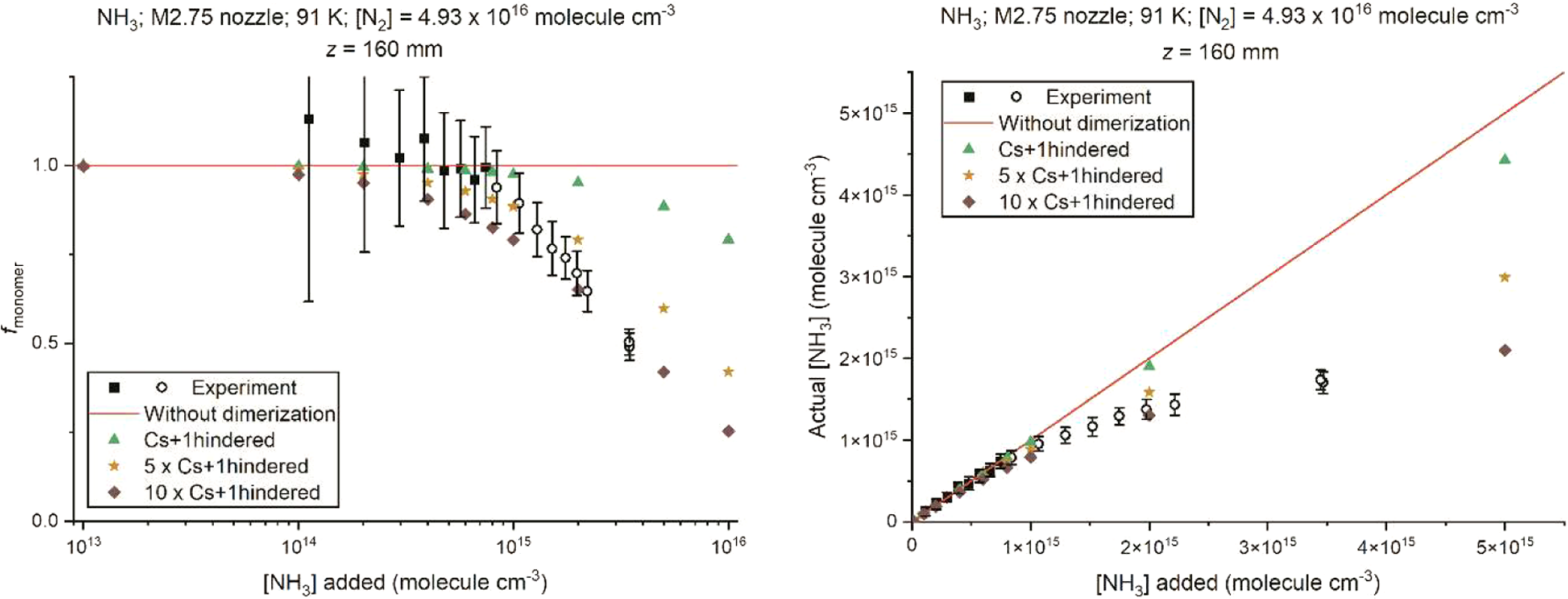
(Left) Measured (using eq 2) and calculated (eq 7) fraction of NH_3_ monomers, *f*_monomer_, and (right) calculated or measured (via NH_2_* fluorescence) concentration of NH_3_ monomers (“Actual”) present plotted against the concentration of NH_3_ added in experiments (which would be the assumed concentration of NH_3_ monomers at the nozzle exit and in the stable flow region if there had been no dimerization) for the M2.75 nozzle in N_2_ (*T* = 91 K, density = 4.93 × 10^16^ molecules cm^–3^ in the uniform flow) at *z* = 160 mm. The *f*_monomer_ = 1 red line in the left panel and the *y* = *x* red line in the right panel mark the case of no dimerization. For the experimental measurements, the closed black symbols are those that are well fitted by a red straight line, whereas the open symbols are where curvature is seen. Calculated values from the “Cs+1hindered” model are shown with green points. The amber and brown points were obtained by artificially increasing the dimerization rate coefficients obtained from the “Cs+1hindered” model by factors of 5 and 10, respectively. The large experimental error at the lowest [NH_3_] in the left panel is due to being close to the limit of detection for NH_2_* fluorescence.

**Figure 8:**
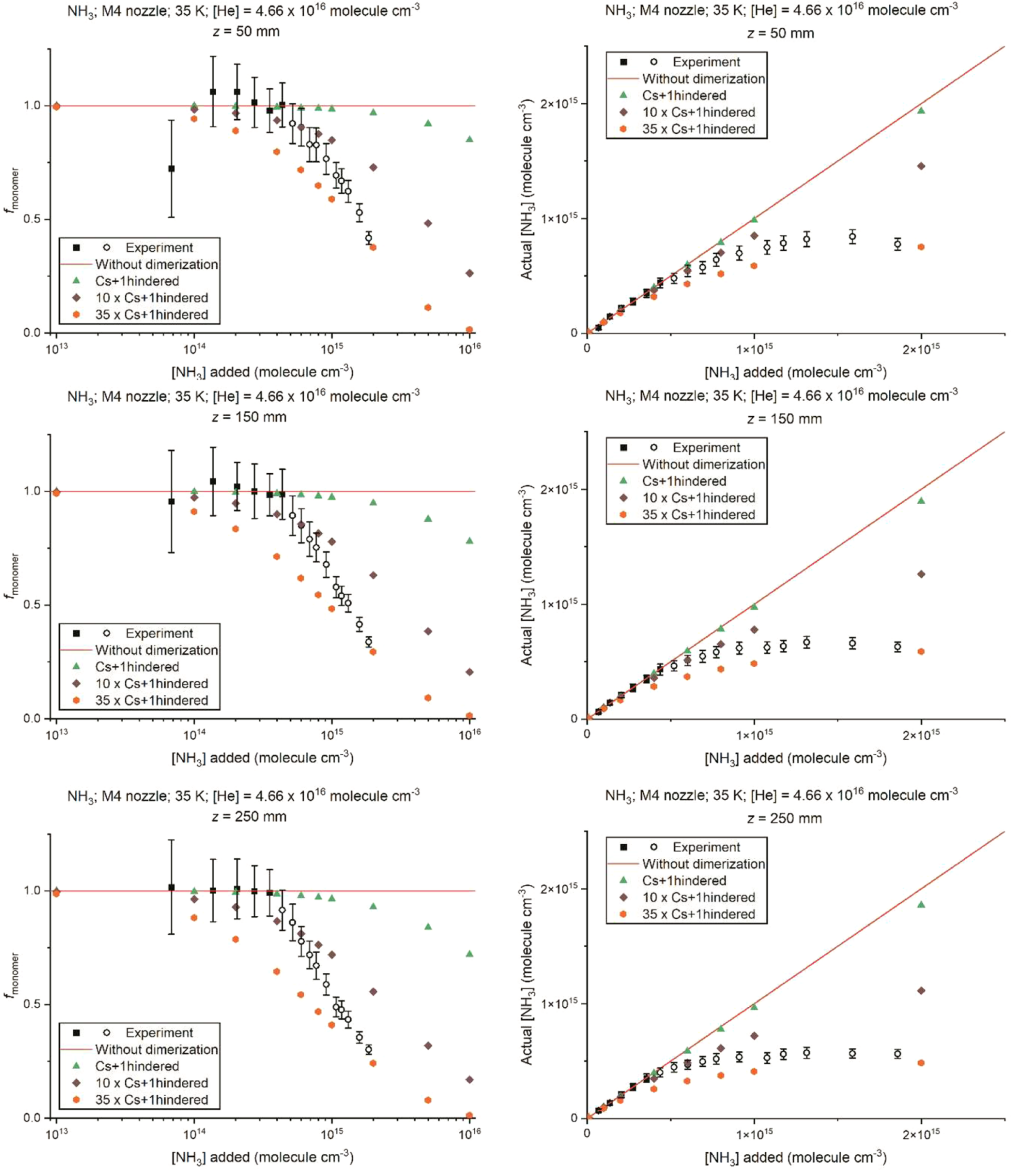
As in Figure 7 but for the M4 nozzle in He (*T* = 35 K, density = 4.66 × 10^16^ molecules cm^–3^ in the uniform flow) (from top to bottom) at *z* = 50, 150, and 250 mm. The brown and orange points were obtained by artificially increasing the dimerization rate coefficients obtained from the “Cs+1hindered” model by a factor of 10 and 35, respectively. The large experimental error and the apparent *f*_monomer_ < 1 for *z* = 50 mm at the lowest [NH_3_] in the left panel is due to being close to the limit of detection for NH_2_* fluorescence.

Figures 7 and 8 both clearly show that the theoretical calculations using the “Cs+1hindered” model, which gives the largest dimerization rate coefficient, *k*_dimer_, of the three calculation models (shown in Figure S14 of the SI), significantly underestimates the formation of NH_3_ dimers at larger added [NH_3_]. To bring the theoretical and experimental results into better agreement at larger added [NH_3_], increasing the value of *k*_dimer_ by a factor of ca. 5–10 is needed for the N_2_ nozzle at 91 K, whereas for the He nozzle at 35 K, an increase in *k*_dimer_ by a factor of ca. 10–35 is needed. However, for both nozzles, this overestimates dimer formation at low [NH_3_] added. We discuss the possible reasons for these discrepancies in the next section. The variation of *f*_dimer_ and *f*_monomer_ versus distance *z* with these increased values of *k*_dimer_ can be found in Figures S22–S23 in the SI.

## 4 Discussion

Figures 7 and 8 show that there is a disagreement between experiment and theory for the variation of *f*_monomer_ with [NH_3_], with theory underestimating the degree of dimerization. A simple increase in the theoretical value of *k*_dimer_ does not completely reproduce the shape of the experimental variation of *f*_monomer_ with [NH_3_]. In the theoretical model, thermal dissociation of the NH_3_ dimer back to the NH_3_ monomers is not considered, but this would overestimate the fraction of dimers formed. Artificially increasing the well depth of the dimer by ∼10 kJ mol^–1^ leads to an increase in *k*_dimer_ and better agreement between the experiment and theory for the loss of NH_3_. However, the relative electronic energies of the NH_3_ monomer and dimer calculated in this work are similar to values reported in previous studies, including those calculated at the UCCSD­(T)-F12a/AVTZ level^63^ of theory, as shown in Table 4, and hence a significant increase in the dimer binding energy does not seem warranted. Some previous theoretical studies reported that both of the optimized C_s_ and C_2h_ dimer structures of the dimer can be located, depending on the level of theory used. Hence, instead of having only one entrance channel available (leading to the C_s_ dimer only for the “Cs” model while to the C_2h_ dimer only for the “C2h” model), the rate coefficient calculations were repeated with both entrance channels active, while dividing *A*_ILT_^∞^ of each channel by 2 to keep the total capture rate coefficient the same. The resulting *k*_dimer_ values are only ∼30% larger than those obtained from the “Cs” model. Hence, just including an extra entrance channel leading to another dimer conformer with a similar well depth cannot increase *k*_dimer_ by an order of magnitude, which is needed to match experiment with theory.

Another factor that could contribute to an underestimation of the predicted *k*_dimer_ and hence fraction of dimers formed is a deficiency in describing the available density of states of the dimer in the transition state theory calculations. As demonstrated by the difference between the “C2h” and “Cs” models, the calculations of the density of states are sensitive to the low vibrational frequency values. These vibrational modes in the dimer typically emerge from the approach of the two monomers. Due to the “floppy” nature of the dimer structure arising from the weak intermolecular interactions, these low frequency values can be subject to large relative errors. Considering the C_s_ dimer structure (Table 3), which is the one with a smaller lowest vibrational frequency value, Table 6 lists the six low-lying vibrational frequencies corresponding to the intermolecular interactions obtained in this work and from previous calculations or modeling.^98,99^ The reported values for the lowest vibrational frequency from previous work are ∼50% of that obtained from the BHandHLYP/aug-cc-pVDZ level in this work, and *k*_dimer_ can be increased by about a factor of 2–4 by replacing the six lowest harmonic frequencies in the “Cs” model with the frequency values from previous work.

**Table 6:** Six Lowest Harmonic Frequencies (cm^–1^) Calculated for the Intermolecular Vibrational Modes of the C_s_ Conformer of the Ammonia Dimer

**method**	**wavenumber** (cm^–1^)
this work^a^	43.6671 101.0479 115.7912 142.3355 253.8584 389.8950
Frisch et al.^98^	20 121 137 166 293 444
Dykstra et al.^99^	27 81 120 160 186 334

a This work: BHandHLYP/aug-cc-pVDZ:^98^ MP2/6–31+G­(d),:^99^ Modeling from molecular mechanics for clusters analysis.

A significant increase in the calculated value of *k*_dimer_ could be achieved by further reducing the lowest vibrational frequency, and indeed, experimental spectroscopic studies show that the lowest observed frequency is ∼13 cm^–1^.^57^ Underrepresentation of the density of states available may result from the harmonic oscillator approximation. Instead of merely taking the lowest vibrational frequency value as a harmonic oscillator, a hindered rotor potential was provided to describe this vibrational mode in the “Cs+1hindered” model (and used for all the *k*_dimer_ calculations shown above), and the resulting *k*_dimer_ is about a factor of 2–4 higher than that of the “Cs” model. With the internal twisting mode with the lowest vibrational frequency described with a hindered rotor potential, the “Cs+1hindered” model should already be the best possible description of the available density of states of the dimer that can be achieved by this work. Further reducing the internal twisting barrier brings negligible change to *k*_dimer_ as lowering the peaks in the hindered rotor potential shown in Figure 2 leads to less than a 1% increase in *k*_dimer_. Hence, a more realistic description of the low-frequency mode (e.g., inclusion of a hindered rotor potential) does promote the efficiency for dimerization, as seen in Figures 7 and 8.

Another factor which controls the values of *k*_dimer_ and hence the loss of monomer is the collision energy transfer parameter, ⟨Δ*E*⟩_down_, defined in eq 4, with values used in the parametrization listed in Table 2 and the variation of ⟨Δ*E*⟩_down_ with *T* for N_2_ and He shown in Figure S3. As shown in Figure 4, *k*_dimer_ is larger for the stronger collider gas N_2_ compared to He. The empirical values used in this work for the calculation of ⟨Δ*E*⟩_down_ are generalized from experimental observations.^92^ While the ordering of the strength as a collider N_2_ > He is correct, considerable uncertainties exist for the use of the parametrization at very low temperatures to calculate the absolute value of ⟨Δ*E*⟩_down_. In order to explore the sensitivity of *k*_dimer_ to the choice of ⟨Δ*E*⟩_down_ for He collider (⟨Δ*E*⟩_*d*own,ref_ = 100 cm^–1^ used above), *k*_dimer_ was calculated for ⟨Δ*E*⟩_down,ref_ = 200, 400, and 1000 cm^–1^. The results are shown in Figure 9, which indicate that *k*_dimer_ follows almost a linear relationship with ⟨Δ*E*⟩_down_, rationalized by the kinetics of the dimerization reaction being at the low-pressure limit. Hence, increasing ⟨Δ*E*⟩_down_ for He to 1000 cm^–1^ will increase *k*_dimer_ by around an order of magnitude, and He would then be considered as a strong collider.^100^ However, for the limited number of reactions that have been studied in a Laval experiment at very low temperatures, and been found to be pressure-dependent, for example, OH + C_2_H_4_,^70,101^ there appears to be no need for such a large increase in the efficiency of the bath gas in transition state theory calculations to match experimental data. However, further experiments to directly validate the parametrization for ⟨Δ*E*⟩_down_ at very low temperatures are needed.

**Figure 9:**
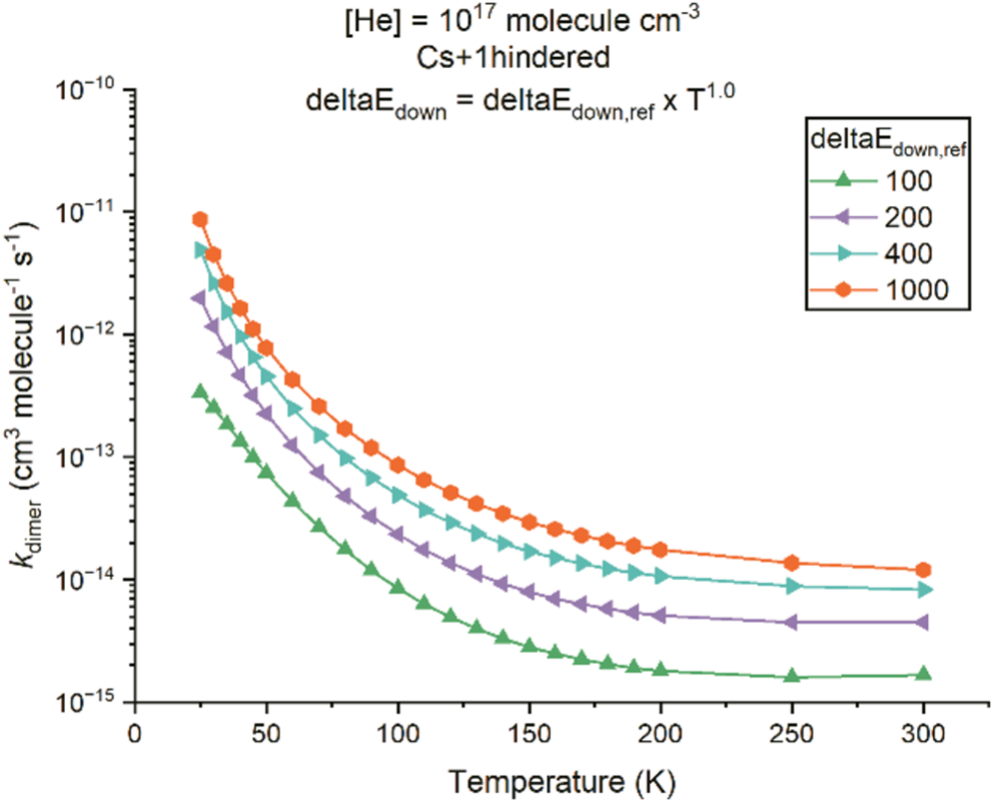
Calculated dimerization rate coefficient, *k*_dimer_, versus temperature for *T* = 25–300 K with a total density of 10^17^ molecules cm^–3^ in He bath gas using the “Cs+1hindered” model with different collisional energy transfer parameters. The temperature-dependent ⟨Δ*E*⟩_down_ is calculated from ${\left\langle \Delta E\right\rangle }_{\mathrm{down}}={\left\langle \Delta E\right\rangle }_{\mathrm{down},\mathrm{ref}}{\left(\frac{T}{298\;K}\right)}^{n}$ , where the legend denotes the ⟨Δ*E*⟩_down,ref_ (in cm^–1^) used, which is varied from 100 cm^–1^ (original value used) to 1000 cm^–1^.

A higher than expected effective energy transfer can be achieved via the chaperone effect, whereby a third-body molecule stabilizes product formation through complexation. Some previously reported examples include the H_2_ molecule in the ionic association of COH^+^ + CO to form (CO)_2_H^+^,^102^ the Ar atom in the recombination reaction of O + O_2_ to form O_3_ ^103^ and S + S_2_ to form S_3_,^104^ and water vapor H_2_O and methanol CH_3_OH in the self-reaction of hydroperoxy radical HO_2_ and methylperoxy radical CH_3_O_2_.^105^ While third-body reactions may further promote ⟨Δ*E*⟩_down_, for the Laval experiments performed to measure the loss of NH_3_ monomers, the gas mixture prepared contains only NH_3_ and the buffer gas, which leaves the system with no other possible choice as the chaperone species. Scattering resonances have been calculated for low-collision cross sections for rotational energy transfer in rotation–inversion transitions in NH_3_ with He and Ar,^106,107^ with an electronic binding energy of NH_3_ with He calculated to be only ∼35 cm^–1^.^107^ However, rate coefficients for collisional relaxation of the bending umbrella vibration are calculated to be very small.^108^ Hence, it seems unlikely that increasing the value of ⟨Δ*E*⟩_down_ alone for the bath gas can bring about the removal of the NH_3_ monomer, as observed in the experiments.

## 5 Conclusions

The kinetics of the dimerization reaction of NH_3_ has been investigated using both experimental and theoretical approaches to provide a quantitative model to predict the production of dimers within the gas expansion inside a Laval nozzle and downstream in the uniform flow region at very low temperatures. Fluorescence from electronically excited NH_2_ (*A* ^2^A_1_), formed from the photolysis of NH_3_, was used to monitor NH_3_ in the flow as a function of added [NH_3_] at several positions downstream of two Laval nozzles, one with a postnozzle uniform flow at 91 K with [N_2_] = 4.93 × 10^16^ molecules cm^–3^ and the other with a postnozzle uniform flow at 35 K with [He] = 4.66 × 10^16^ molecules cm^–3^. No dimerization, corresponding to a fraction of the NH_3_ monomer remaining in the flow, *f*_monomer_ = 1, was experimentally observed up to ∼ [NH_3_] = 1 × 10^15^ molecules cm^–3^ for 160 mm downstream of the 91 K N_2_ nozzle, and up to ∼5 × 10^14^ molecules cm^–3^ for 150 mm downstream of the 35 K He nozzle. Dimerization, corresponding to *f*_monomer_ < 1, was observed at higher added [NH_3_], being more pronounced at the lower flow temperature in He.

Electronic structure calculations of the optimized geometric structures, relative energies, and ro-vibrational properties of the dimer, performed at the BHandHLYP/aug-cc-pVDZ level, only identified the C_s_ conformer of the NH_3_ dimer. At the M06-2X/aug-cc-pVTZ level of theory, only the C_2h_ dimer was identified. The refined binding energies of the C_s_ and C_2h_ dimers at the CCSD­(T)/aug-cc-pVTZ level are about the same, with ZPVE corrected values with respect to the monomers of −7.52 and −7.33 kJ mol^–1^, respectively, similar to previously reported values. Using the calculated energies, the open-source statistical rate theory software package MESMER was used to calculate the NH_3_ dimerization rate coefficient, *k*_dimer_, for both He and N_2_ bath gases. The calculated *k*_dimer_ displayed the expected negative dependence upon temperature and the positive dependence upon pressure of the bath gas. *k*_dimer_ was sensitive to the values of the low vibrational frequencies of the dimer and to the inclusion of a hindered rotor potential for the C_s_ dimer. The calculation model “Cs+1hindered” gave the largest value of *k*_dimer_, as the density of states of the dimer is increased by a smaller lowest vibrational frequency value from the hindered rotor potential. *k*_dimer_ is larger for N_2_ than for He owing to the larger energy removed from the internally excited dimer per collision. The fraction of NH_3_ remaining in the flow, *f*_monomer_, was calculated as a function of the NH_3_ concentration for several distances downstream of the exit of the Laval nozzles. Unlike in the experiments, *f*_monomer_ could also be calculated within the body of the nozzle itself. The axial velocity, temperature, and number density profiles within the 91 and 35 K nozzles were first determined in two ways. In the first, a scaling was performed of the profile for a previous nozzle at 52 K assuming an isentropic core, and in the second, by performing a detailed computational fluid dynamics simulation. These profiles agreed very well, providing a validation of the scaling method. Profiles of *k*_dimer_ and hence *f*_monomer_ were then calculated from the sonic point within the nozzle to the nozzle exit. Dimerization occurs both within the nozzle itself and in the stable flow region, with the calculations showing that more occurs within the nozzle per unit distance traversed.

The measured and calculated *f*_monomer_ values were compared as a function of added NH_3_ for varying distances outside the nozzle in the stable, uniform region. For the “Cs+1hindered” model, the calculations substantially underestimated the degree of dimerization, indicating a significant underprediction of the value of *k*_dimer_. To match observations, the calculated *k*_dimer_ needs to be increased by a factor of ca. 5–10 for the 91 K nozzle in N_2_, and by a factor of ca. 10–35 for the 35 K nozzle in He. Several factors that can increase the *k*_dimer_ were considered. The presence of two entrance channels forming two conformers of the dimer increases the *k*_dimer_. Significantly increasing the well depth of the dimer does not seem warranted, as the calculated value in this work agrees well with previous studies. The inclusion of a hindered potential for the lowest vibrational frequency mode of the dimer did increase the density of states and hence the *k*_dimer_, showing that the presence of torsional modes can further increase the density of states. The energy removed per collision may also be underestimated at very low temperatures, with the empirical parametrization used to describe ⟨Δ*E*⟩_*down*_ being subject to considerable uncertainty for low collision energies, where nonstatistical effects may need to be considered. Further experimental and theoretical studies are needed to investigate energy transfer in very low-energy collisions. It is also assumed that only dimerization causes loss of the NH_3_ monomer, whereas formation of NH_3_ trimers or higher oligomers may occur, which could influence the magnitude and shape of *f*_monomer_ with [NH_3_].

## Supplementary Material



## Notes

Additional experimental results for other nozzles and conditions not covered in the main paper, geometric coordinates, vibrational energy levels and rotational constants obtained from the theoretical calculations, collision theory calculations of the dimer rate coefficient, and rate coefficient calculations as a function of temperature and pressure using different levels of theory to provide inputs to MESMER; computational fluid dynamic calculations of the profiles of temperature, density, and Mach number; and calculations of the fraction of NH_3_ monomer remaining for a wider range of conditions and nozzles at different levels of theory (Sections S1–S14, Figures S1–S23, Tables S1–S11) (PDF)
