# Supplementary material for: Experimental and Theoretical Study of the Kinetics of Dimerization of Ammonia at Low Temperatures
Source: J Phys Chem A. 2025 Jul 1;129(28):6289–305. doi: 10.1021/acs.jpca.5c03008 (PMC12278223; doi:10.1021/acs.jpca.5c03008)
Supplement: Supplementary file 1 [file jp5c03008_si_001.pdf]

Supporting Information

Experimental and Theoretical Study of the Kinetics of Dimerization of Ammonia at Low Temperatures

Lok Hin Desmond Li [a], Kevin M. Douglas [a], Ffion Hall [a], Alice Kirker [a], Luke Driver [b], Gregory N. de Boer [c], Nikil Kapur [c], Julia H. Lehman [d], Mark A. Blitz [a, e], Dwayne E. Heard [a] *

[a] *School of Chemistry, University of Leeds, Leeds, LS2 9JT, UK*

[b] *EPSRC CDT in Fluid Dynamics, School of Computing, University of Leeds, Leeds, LS2 9JT, UK*

[c] *School of Mechanical Engineering, University of Leeds, Leeds, LS2 9JT, UK*

[d] *School of Chemistry, University of Birmingham, Edgbaston, Birmingham, B15 2TT, UK*

[e] *National Centre for Atmospheric Science (NCAS), University of Leeds, Leeds, LS2 9JT, UK*

* Corresponding author. Email: d.e.heard@leeds.ac.uk

S1. NH_2^* Fluorescence signals from experiments

Experimental measurements for the M4 in He nozzle at z = 50 mm, 150 mm and 250 mm downstream of the nozzle exit are shown below in Figure S1 while those for the M2.75 in N_2 nozzle are shown in Figure 1 in the main text.

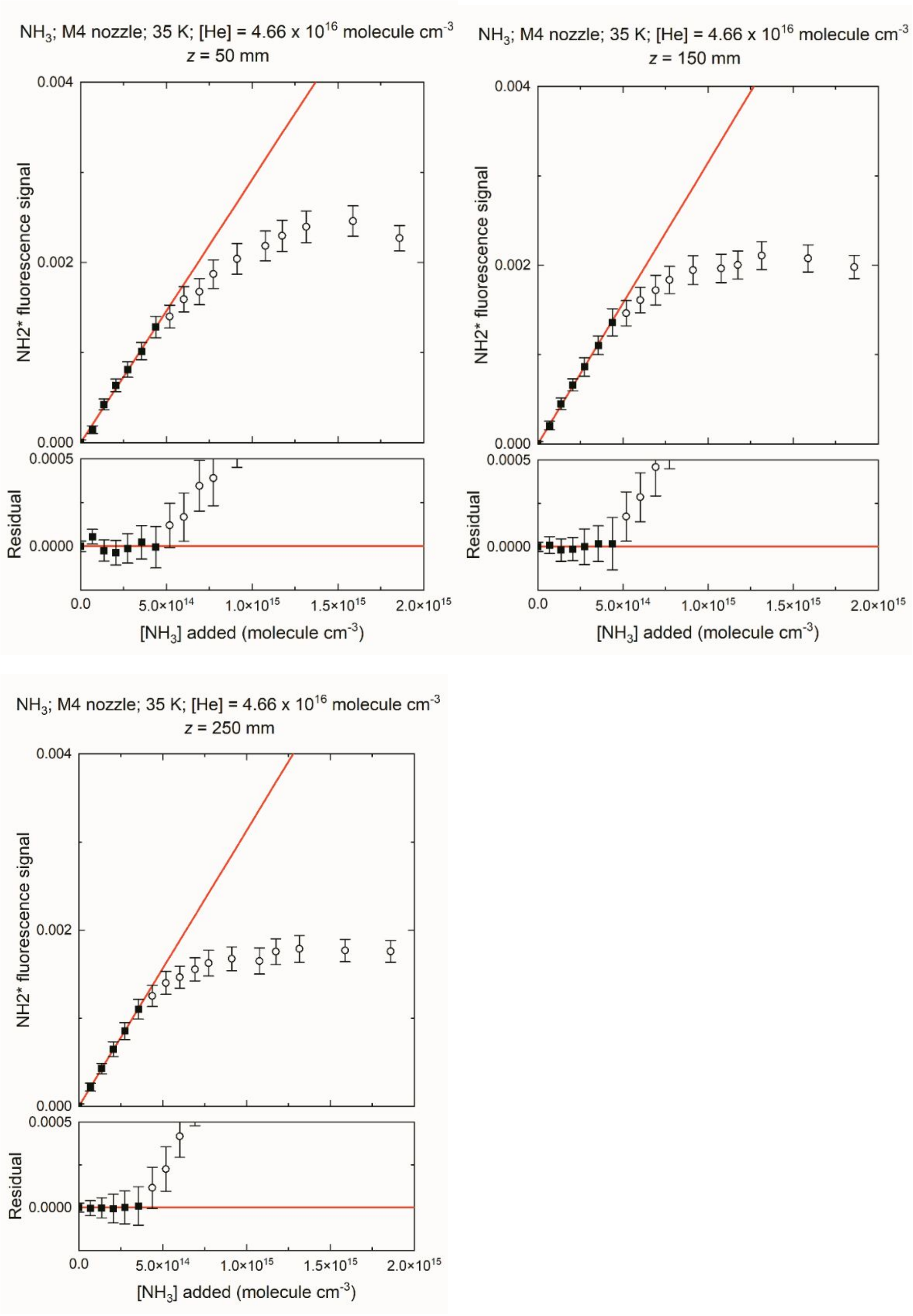

Figure S1. The variation of the NH_2^* fluorescence signal (arb units) z = 50 mm (top left), 150 mm (top right) and 250 mm (bottom) downstream from the nozzle exit versus [NH_3] added to the flow for the M4 nozzle with He bath gas. The [NH_3] is the concentration at the nozzle exit (z=0) calculated from the mole fraction of NH_3 used to make up the gas mixture and the total density of the flow. At low [NH_3] the fluorescence signal is linear with [NH_3] added (closed squares) with a straight line fitted to the closed symbols shown in red. At high [NH_3] there is downward curvature from the fitted line (open circles). Residuals from the best-fit line (bottom part of each panel). The error bars represent 1σ error in the fluorescence signal.

S2. *Ab initio* calculations using Gaussian[1]

Table S1. Electronic energies (Hartree) of the NH_3 monomer and dimer C_s and C_{2h} conformers.

Molecule	BHandHLYP/ aug-cc-pVDZ	CCSD(T)/ aug-cc-pVTZ// BHandHLYP/ aug-cc-pVDZ	M06-2X/ aug-cc-pVTZ	CCSD(T)/ aug-cc-pVTZ// M06-2X/ aug-cc-pVTZ
NH_3	–56.5305370620	–56.480457169	–56.5529642916	–56.480527651
$(NH_3)_2$ C_s	–113.065935248	–112.96610227		
$(NH_3)_2$ C_{2h}			–113.111001119	–112.96618540

Table S2. Zero-point vibrational energies (ZPVE) (Hartree) of the NH_3 monomer and dimer C_s and C_{2h} conformers.

Molecule	Unscaled BHandHLYP/ aug-cc-pVDZ	Scaled BHandHLYP/ aug-cc-pVDZ (scaling factor: 0.9589)	Unscaled M06-2X/ aug-cc-pVTZ	Scaled M06-2X/ aug-cc-pVTZ (scaling factor: 0.956)
NH_3	0.035216	0.033768622	0.034461	0.032944716
$(NH_3)_2$ C_s	0.072855	0.067537245		
$(NH_3)_2$ C_{2h}			0.071368	0.068227808

Table S3. Optimized (BHandHLYP/aug-cc-pVDZ) Cartesian coordinates of the NH_3 monomer and dimer C_s conformer.

Molecule	Atom, (*x,y,z*) coordinates			
NH_3	N	-0.03910700	0.12702000	0.00000200
	H	0.33096400	-0.81130700	-0.00000100
	H	0.33098200	0.59617900	0.81261100
	H	0.33097200	0.59617900	-0.81261200
$(NH_3)_2$ C_s	N	1.56915900	0.00000000	-0.02036900
	H	1.56919200	-0.00000100	0.98853600
	H	2.08759000	-0.81189200	-0.31897200
	H	2.08759000	0.81189200	-0.31897100
	N	-1.70810500	0.00000000	0.09956000
	H	-0.72160300	0.00000000	-0.12918500
	H	-2.12331400	-0.81057700	-0.33386900
	H	-2.12331300	0.81057900	-0.33386700

Table S4. Optimized (M06-2X/aug-cc-pVTZ) Cartesian coordinates of the NH_3 monomer and dimer C_{2h} conformer.

Molecule	Atom, (*x,y,z*) coordinates			
NH_3	N	-0.04248000	0.12703500	0.00000200
	H	0.33209300	-0.81320500	-0.00000100
	H	0.33210500	0.59712000	0.81427900
	H	0.33209300	0.59712000	-0.81428000
$(NH_3)_2$ C_{2h}	N	1.56896700	0.00000000	0.08174500
	H	0.85899600	0.00000200	-0.64293900
	H	2.15309400	0.81345800	-0.06518300
	H	2.15308900	-0.81346100	-0.06518300
	N	-1.56896700	0.00000000	-0.08174400
	H	-0.85899600	0.00000200	0.64293900
	H	-2.15309400	0.81345800	0.06518300
	H	-2.15308900	-0.81346100	0.06518300

Table S5. Unscaled harmonic vibrational frequencies (cm^{-1}) of the NH_3 monomer and C_s dimer conformer (BHandHLYP/aug-cc-pVDZ).

Molecule	Wavenumber (cm^{-1})
NH_3	1043.8405 1693.3273 1693.3350 3587.4683 3720.0774 3720.1032
$(NH_3)_2$ C_s	43.6671 101.0479 115.7912 142.3355 253.8584 389.8950 1075.8770 1085.9064 1687.6544 1694.5338 1698.3135 1719.3056 3553.9956 3585.5179 3684.2928 3714.5168 3714.9470 3718.0042

Table S6. Unscaled harmonic vibrational frequencies (cm^{-1}) of the NH_3 monomer and dimer C_{2h} conformer (M06-2X/aug-cc-pVTZ).

Molecule	Wavenumber (cm^{-1})
NH_3	1031.9317 1659.0495 1659.6365 3508.5788 3633.1568 3634.3644
$(NH_3)_2$ C_{2h}	80.7821 83.0255 101.0968 146.3276 222.6985 441.7019 1047.6947 1078.0634 1646.3504 1663.6986 1665.3240 1675.0643 3491.6979 3495.5942 3613.2567 3613.3118 3630.6236 3630.6945

Table S7. Rotational constants of NH_3 monomer and C_s dimer conformer (BHandHLYP/aug-cc-pVDZ).

Molecule	Rotational constants	
NH_3	(in GHz)	302.33802 302.33769 189.84799
	(in cm^{-1})	10.078 10.078 6.328
$(NH_3)_2$ C_s	(in GHz)	120.20348 5.17083 5.11319
	(in cm^{-1})	4.007 0.172 0.170

Table S8. Rotational constants of the NH_3 monomer and C_{2h} dimer conformer (M06-2X/aug-cc-pVTZ).

Molecule	Rotational constants	
NH_3	(in GHz)	299.87944 299.86305 189.07759
	(in cm^{-1})	9.996 9.995 6.303
$(NH_3)_2$ C_{2h}	(in GHz)	137.94855 5.60569 5.50365
	(in cm^{-1})	4.598 0.187 0.183

S3. Calculations of the collision limit rate coefficient, k_{coll}, to be used as the Inverse Laplace Transform (ILT) input parameters in MESMER

From the classical capture theory,[2-4] the collision limit $k_{\text{coll}}(T)$ for NH_3 dimerization is given by:

$$k_{\text{coll}}(T) = 1.353\pi\left(\frac{2C_6}{k_B T}\right)^{\frac{1}{3}}\left(\frac{8k_B T}{\pi\left(\frac{m_{\text{NH}_3}}{2}\right)}\right)^{\frac{1}{2}} \tag{S1}$$

where k_B is the Boltzmann constant and m_{NH_3} is the mass of NH_3. C_6 is the coefficient that describes the magnitude of attractive forces and when multiple sources of attractive forces are considered, C_6 can be represented as a sum of coefficients from each component. Possible sources of attractive forces include dipole-dipole $D-D$, dipole-induced dipole $D-iD$, and dispersion forces $Disp$, where their respective coefficients can be calculated from:

$$C_6^{D-D} = \frac{2}{3}\left(\frac{\mu_{\text{NH}_3}^4}{k_B T(4\pi\varepsilon_0)^2}\right) \tag{S2}$$

$$C_6^{D-iD} = \frac{\mu_{\text{NH}_3}^2 \alpha_{\text{NH}_3}}{2\pi\varepsilon_0} \tag{S3}$$

$$C_6^{Disp} = \frac{3}{4}\alpha_{\text{NH}_3}^2 I_{\text{NH}_3} \tag{S4}$$

with ε_0 being the permittivity of free space. μ_{NH_3}, α_{NH_3}, and I_{NH_3} are the dipole moment, the polarizability, and the ionization energy of NH_3, which are properties that can be obtained from electronic structure calculations, with the results as shown in Table S9. The contributions from the dipole-dipole forces on k_{coll} follow the relationship of $T^{-\frac{1}{6}}$, while from the dipole-induced dipole and dispersion forces follow the relationship of $T^{\frac{1}{6}}$. The contributions from the dipole-dipole forces dominate at low temperature[5] and to demonstrate this, k_{coll} was computed for two scenarios: C_6 as a sum of C_6^{D-D}, C_6^{D-iD} and C_6^{Disp}; and C_6 consisting of C_6^{D-D} only. Using the parameters obtained at the same level of theory, the dipole-dipole forces contribute to >99 % of k_{coll} below 15 K, >90 % below 200 K, and >85% below 300 K as shown in Figure S2. In this work, $k_{coll}(T)$ was taken as the high-pressure limiting coefficient $k^{\infty}(T)$, which in the MESMER input takes the expression of:

$$k^{\infty}(T) = A_{\mathrm{ILT}}^{\infty}\left(\frac{T}{298\ \mathrm{K}}\right)^{n_{\mathrm{ILT}}^{\infty}} \quad \text{(S5)}$$

By assuming C_6 consisting of C_6^{D-D} only, which was done in this work for the subsequent rate coefficient calculations, the ILT parameters $A_{\mathrm{ILT}}^{\infty}$ and $n_{\mathrm{ILT}}^{\infty}$ can be easily obtained ($n_{\mathrm{ILT}}^{\infty} = -\frac{1}{6}$ and $A_{\mathrm{ILT}}^{\infty}$ computed from the dipole moment) as $k_{coll}(T)$ becomes:

$$k_{\mathrm{coll}}(T) = 1.353\pi\left(\frac{\mu_{\mathrm{NH_3}}^4}{12k_{\mathrm{B}}^2\pi^2\varepsilon_0^2}\right)^{\frac{1}{3}}\left(\frac{8k_{\mathrm{B}}}{\pi\left(\frac{m_{\mathrm{NH_3}}}{2}\right)}\right)^{\frac{1}{2}}T^{-\frac{1}{6}} \quad \text{(S6)}$$

This approximation is valid as shown in Figure S2, in particular k_{coll} obtained from including C_6^{D-D} only is almost exactly the same as that from the sum of C_6^{D-D}, C_6^{D-iD} and C_6^{Disp} at the lowest temperature.

Table S9. Some physical constants of NH_3 obtained from electronic structure calculations which are used for the calculation of k_{coll}.

**Property**	**Level of theory**	**Value**
Dipole moment μ (Debye)	CCSD(T)/aug-cc-pVTZ// BHandHLYP/aug-cc-pVDZ	1.5836
	CCSD(T)/aug-cc-pVTZ// M06-2X/aug-cc-pVTZ	1.5936
Polarizability α (cm^{-3})	BHandHLYP/aug-cc-pVDZ	1.97×10^{-24}
	M06-2X/aug-cc-pVTZ	2.05×10^{-24}
Ionization energy I (eV)	CCSD(T)/aug-cc-pVTZ// BHandHLYP/aug-cc-pVDZ	10.102
	CCSD(T)/aug-cc-pVTZ// M06-2X/aug-cc-pVTZ	10.099

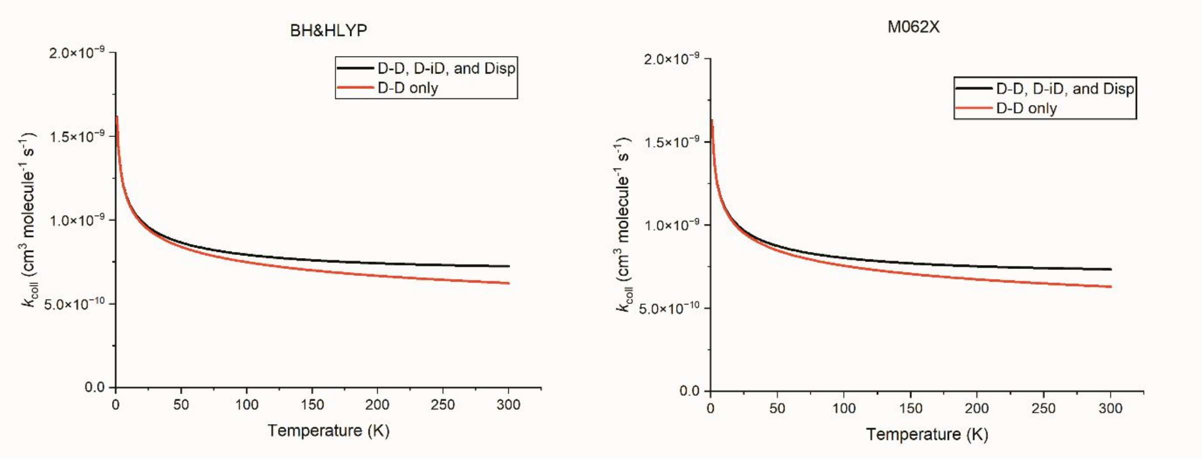

Figure S2. Calculated k_{coll} for the approach of two NH_3 molecules as a function of temperature by considering (black) dipole-dipole $D-D$, dipole-induced dipole $D-iD$ and dispersion forces $Disp$; and (red) dipole-dipole $D-D$ forces only, using results from electronic structure calculations (left) BH&HLYP: μ, I: CCSD(T)/aug-cc-pVTZ//BHandHLYP/aug-cc-pVDZ, α: BHandHLYP/aug-cc-pVDZ; (right) M062X: μ, I: CCSD(T)/aug-cc-pVTZ//M06-2X/aug-cc-pVTZ, α: M06-2X/aug-cc-pVTZ).

S4. Average energy removed per collision, $\langle \Delta E \rangle_{down}$, used in MESMER calculations

To model the pressure dependence of the reaction, the Lennard-Jones ε and σ parameters of the bath gases[6] and the average energy removed per collision by different bath gases are required. Experimental values of the Lennard-Jones parameters of the bath gases reported by Gilbert and Smith,[7] which is listed in Table S10, are used.

Table S10. Values of the Lennard-Jones parameters for different bath gases. Taken from Gilbert and Smith.[7]

Bath gas	ε (K)	σ (Å)
He	10	2.55
N_2	48	3.9

For the probability of a given energy being transferred per collision, the exponential-down model[8] was used in this work, where the probability is derived from the average energy transferred in each deactivating collision $\langle \Delta E \rangle_{down}$. In MESMER, an empirical approach is used, with the temperature dependent $\langle \Delta E \rangle_{down}$ modelled as:

$$\langle \Delta E \rangle_{down} = \langle \Delta E \rangle_{down,ref} \left(\frac{T}{298\ \text{K}} \right)^{n} \quad \text{(S7)}$$

where $\langle\Delta E\rangle_{down,ref}$ and n are the input parameters. The empirical values of $\langle\Delta E\rangle_{down,ref}$ and n used for different bath gases are listed in Table 2 in the main text and the plot of $\langle\Delta E\rangle_{down}$ as a function of temperature for N_2 and He bath gas is shown below in Figure S3.

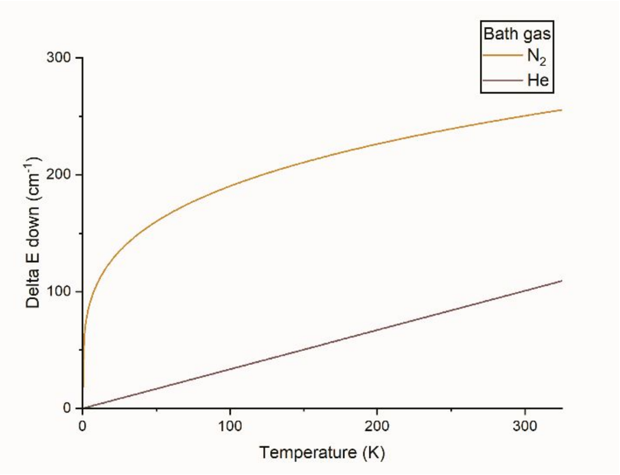

Figure S3. Variation of $\langle\Delta E\rangle_{down}$ as a function of temperature using the parameters given in Table 2 of the main text.

S5. Calculated dimerization rate coefficient, k_{dimer}, versus temperature at various total number densities with different bath gases using different calculation models

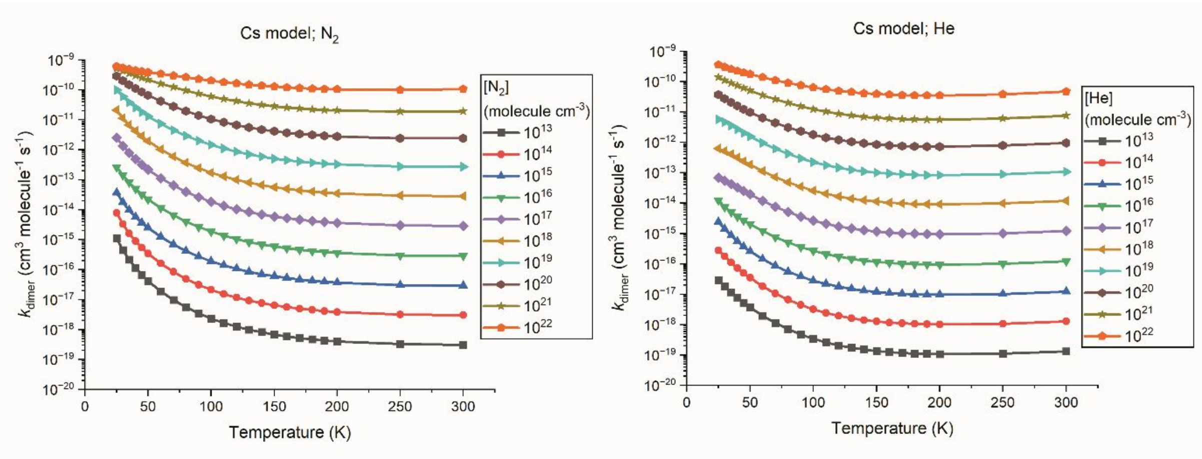

Figure S4. Calculated rate coefficient for NH_3 dimerization, k_{dimer}, versus temperature in the range of 25–300 K at various total number densities using the "Cs" model. The legend denotes the number densities from 10^{13}–10^{22} molecule cm^{-3}. The bath gases used are (left) N_2 and (right) He respectively.

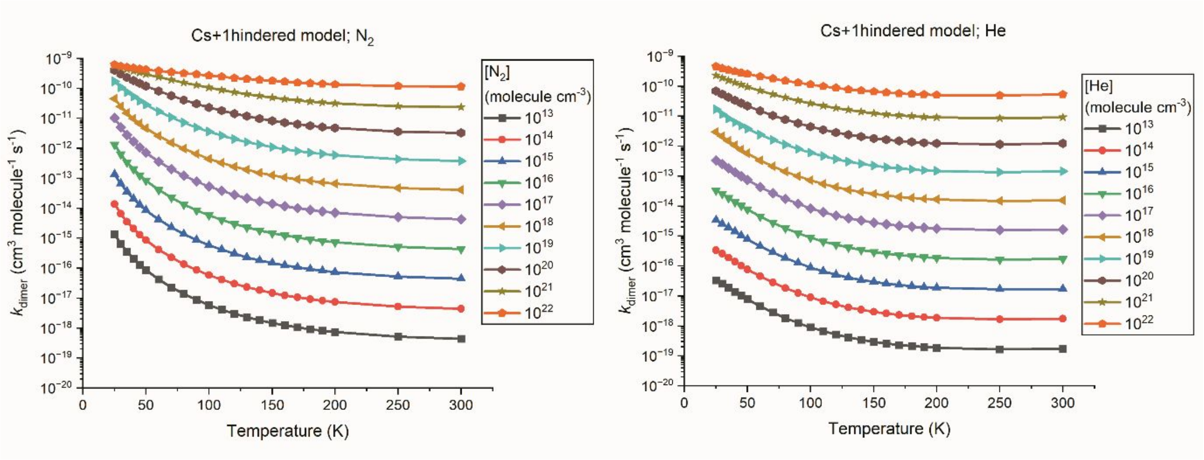

Figure S5. Calculated rate coefficient for NH_3 dimerization, k_{dimer}, versus temperature in the range of 25–300 K at various total number densities using the "Cs+1hindered" model. The legend denotes the number densities from 10^{13}–10^{22} molecule cm^{-3}. The bath gases used are (left) N_2 and (right) He respectively.

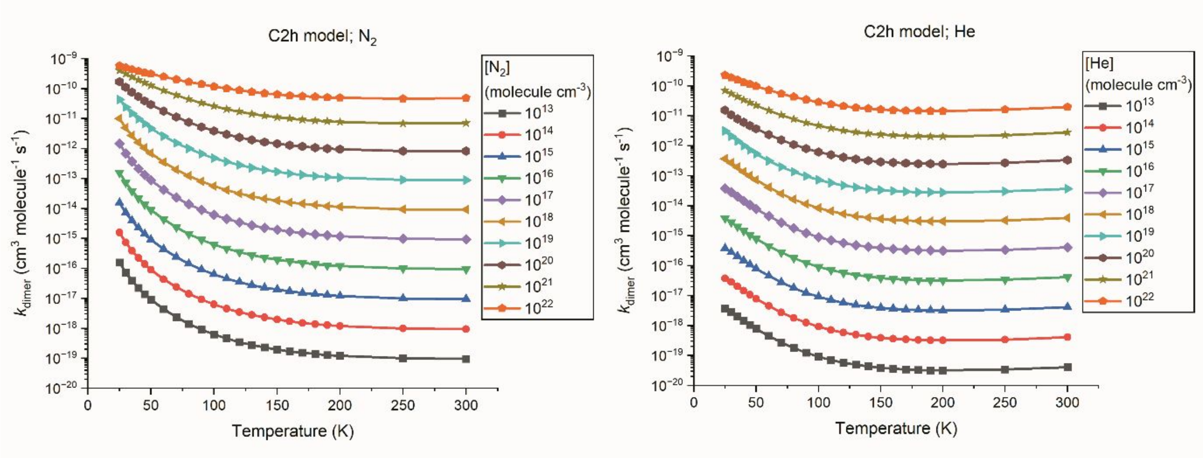

Figure S6. Calculated rate coefficient for NH_3 dimerization, k_{dimer}, versus temperature in the range of 25–300 K at various total number densities using the "C2h" model. The legend denotes the number densities from 10^{13}–10^{22} molecule cm^{-3}. The bath gases used are (left) N_2 and (right) He respectively.

S6. Density of states of the NH_3 dimer

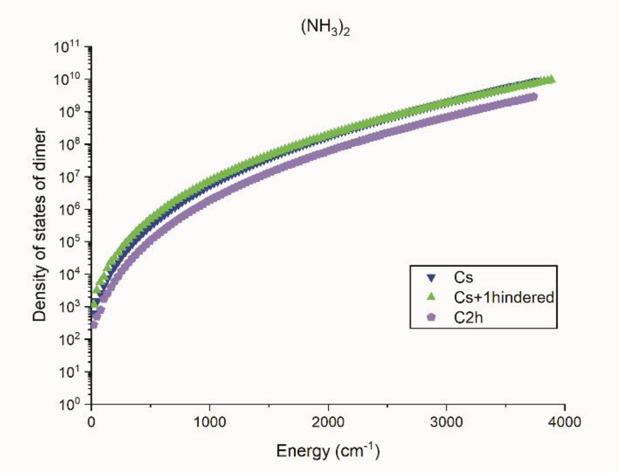

Figure S7. Grain rovibronic density of states of the NH_3 dimer against the grain energy using three different calculation models (blue: "Cs", green: "Cs+1hindered", purple: "C2h").

S7. Temperature, number density and velocity profiles of the nozzles

Figure S8 shows the profiles of temperature, total number density, Mach number and flow speed within (inside) the 52 K Laval nozzle in N_2, taken from Antiñolo et al.[9] $z = 0$ is the nozzle exit (see Figure 3 of the main paper).

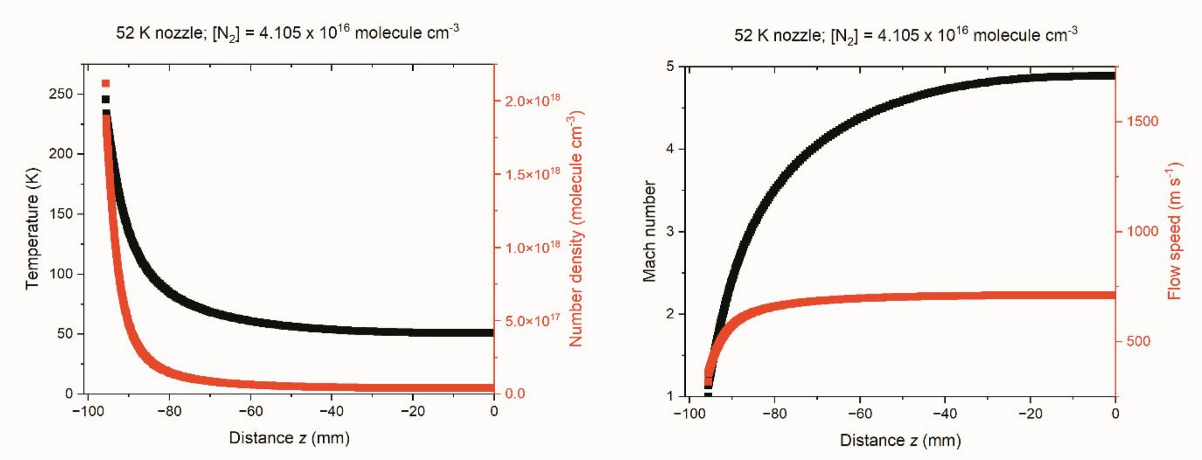

Figure S8. (Left) Temperature (black line) and number density (red line) profiles, and (right) Mach number (black line) and flow speed (red line) profiles within the 52 K nozzle in N_2 from Antiñolo et al.[9] from the sonic point to the nozzle exit ($z = 0$).

Figure S9 shows the scaled profiles of temperature, total number density, Mach number and flow speed within (inside) for the M2.75 nozzle in N_2. $z = 0$ is the nozzle exit (see Figure 3 of main paper).

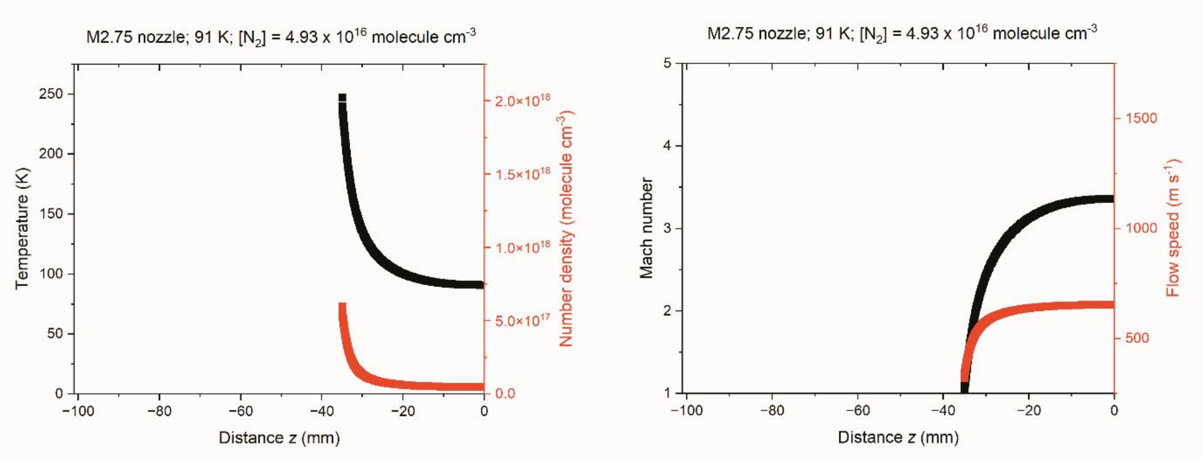

Figure S9. (Left) Scaled temperature (black line) and number density (red line) profiles, and (right) scaled Mach number (black line) and flow speed (red line) profiles within the M2.75 nozzle in N_2. The nozzle exit is at $z = 0$.

Figure S10 shows the scaled profiles of temperature, total number density, Mach number and flow speed within (inside) the M4 nozzle in He. $z = 0$ is the nozzle exit (see Figure 3 of main paper).

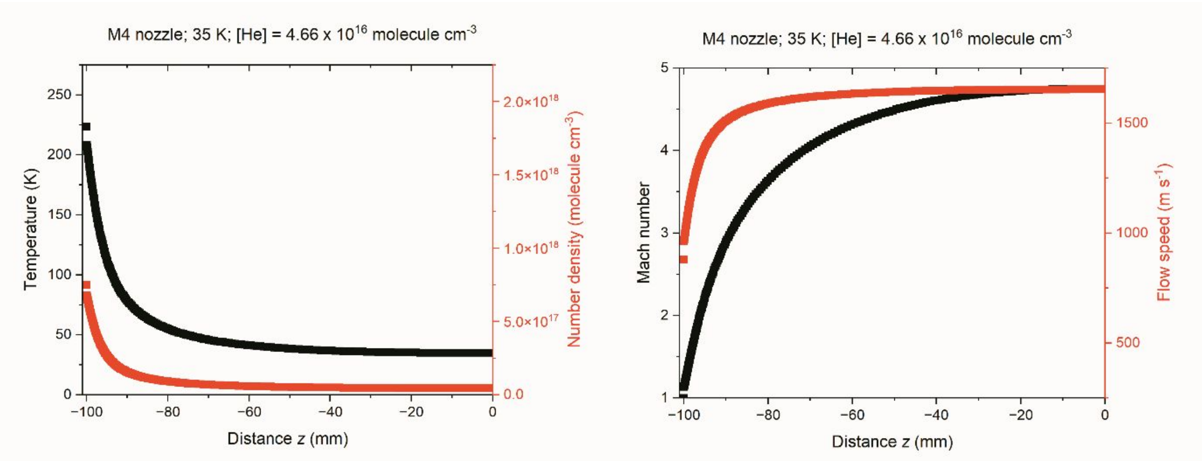

Figure S10. (Left) Scaled temperature (black line) and number density (red line) profiles, and (right) scaled Mach number (black line) and flow speed (red line) profiles within the M4 nozzle in He. The nozzle exit is at $z = 0$.

S8. Validation of the scaled profiles using computational fluid dynamics

In order to validate the scaling procedure used to generate the profiles shown in Figures S9 (M2.75 N_2 nozzle) and S10 (M4 He nozzle) from those shown in Figure S8 for the 52 K nozzle from Antiñolo et al.,[9] computational fluid dynamics calculations were performed. A computational fluid dynamics calculation has been performed for a Mach 2.25 Laval nozzle in N_2 and is reported in Driver et al.[10] A scaling from the 52 K nozzle profiles shown in S8 was performed using the method described in Section 2.2.3, with the parameters of T and total density at the exit of the nozzle constrained using measurements from the laboratory using a Pitot tube. Details of the M2.25 nozzle are Mach number M at exit = 3.02, temperature T at exit = 106 K, number density n at exit = 6.70×10^{16} molecule cm^{-3}. The results from the computational fluid dynamics calculations span the reservoir region upstream of the Laval nozzle, the nozzle itself and the uniform flow region downstream of the nozzle, starting at a distance z of –51.6 mm, where the first 10 mm corresponds to the reservoir and, z of –41.6 mm to $z = 0$ is within the nozzle itself, and $z > 0$ is downstream of the nozzle exit. The scaling was performed for distance of 30 mm from z = - 30 mm to z= 0 mm, The comparison between the Mach number profiles, temperature profiles and number density profiles can be found in Figures S11 – S13, respectively, showing generally good agreement both in shape and magnitude.

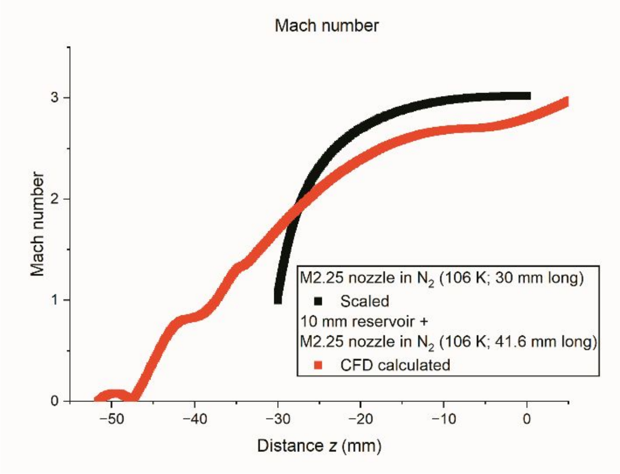

Figure S11. Calculated Mach number profiles for the M2.25 nozzle in N_2 obtained from the scaling method (black) and computational fluid dynamics calculations (red).

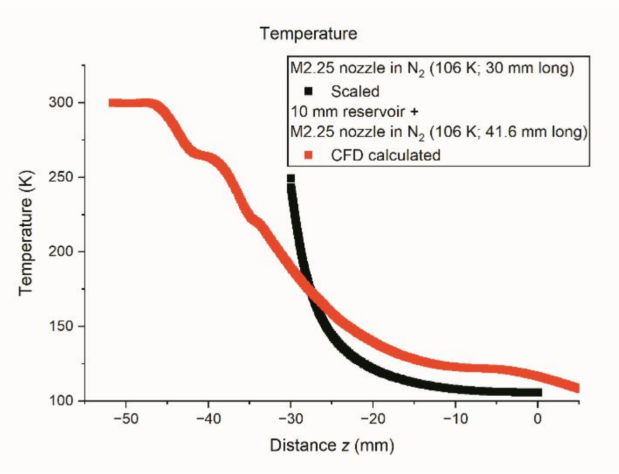

Figure S12. Calculated temperature profiles for the M2.25 nozzle in N_2 obtained from the scaling method (black) and computational fluid dynamics calculations (red).

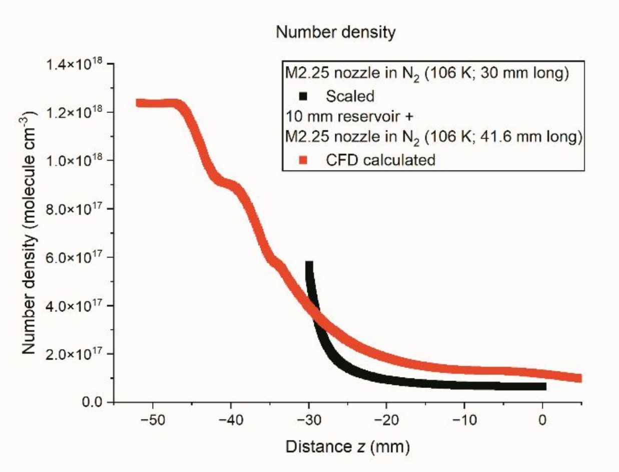

Figure S13. Calculated number density profiles for the M2.25 nozzle in N_2 obtained from the scaling method (black) and computational fluid dynamics calculations (red).

S9. Calculated rate coefficients for NH_3 dimerization, k_{dimer}, versus distance z in the nozzles using different models to calculate the density of states of the dimer.

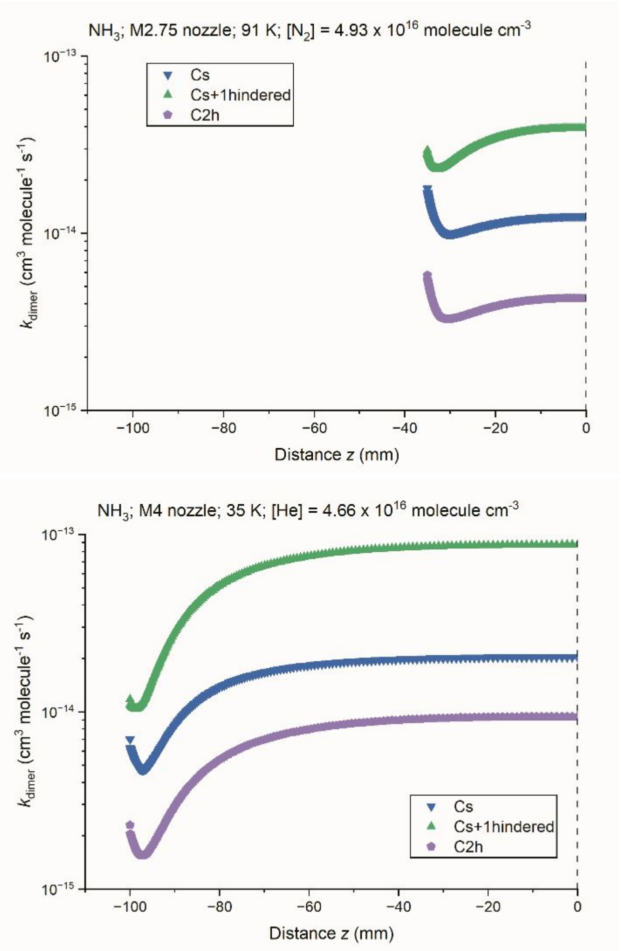

Figure S14. Calculated rate coefficients for NH_3 dimerization, k_{dimer} as a function of flow distance z within (top) the M2.75 $T = 91$ K nozzle in N_2 and (bottom) the M4 $T = 35$ K nozzle in He (vertical dashed line at $z = 0$ is the nozzle exit) using three different calculation models (blue: "Cs", green: "Cs+1hindered", purple: "C2h"). The M4 nozzle is considerably longer than the M2.75 nozzle.

S10. MESMER calculation of the thermal dissociation lifetime of the NH_3 dimer within the Laval nozzles.

For the calculations of the fraction of NH_3 that exists as either a monomer or in dimeric form, using equations (E7) and (E8) it is assumed that Δt is small enough such that the stabilized dimers remain in dimer form when travelling down the flow, rather than dissociating back to the monomer. The thermal dissociation lifetime of the stabilized NH_3 dimers via collision with the bath gas (the reverse of reaction (R1)) as a function of distance within the M2.75 nozzle in N_2 and M4 nozzle in He were calculated using MESMER are shown in Figure S15, together with the travelling time of the gas flow from one data point to the subsequent one (of the 248 data points of the nozzle profiles). While the lifetime of the dimers is longer than the travelling time in the later portion of the nozzle, the lifetime is approximately the same order of magnitude as the travelling time in the earlier portion. Thus, it is expected that there should be a larger overestimation in f_{dimer}, particularly for the first 5 mm for the M2.75 nozzle in N_2.

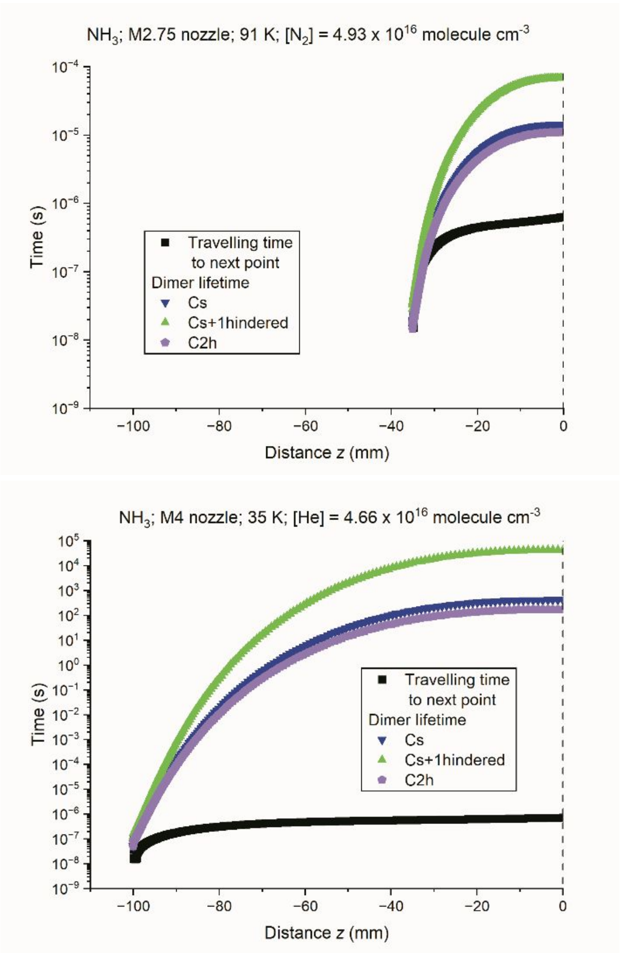

Figure S15. Lifetime of the stabilized dimers of NH_3 as a function of flow distance z within (top) the M2.75 nozzle in N_2 and (bottom) the M4 nozzle in He (vertical dashed line at $z = 0$ is the nozzle exit) using three different calculation models (blue: "Cs", green: "Cs+1hindered", purple: "C2h"). Plotted together is the travelling time of the gas flow from one data point to the subsequent data point (black).

S11. f_{dimer} versus distance z for various added NH_3 concentrations using different calculation models.

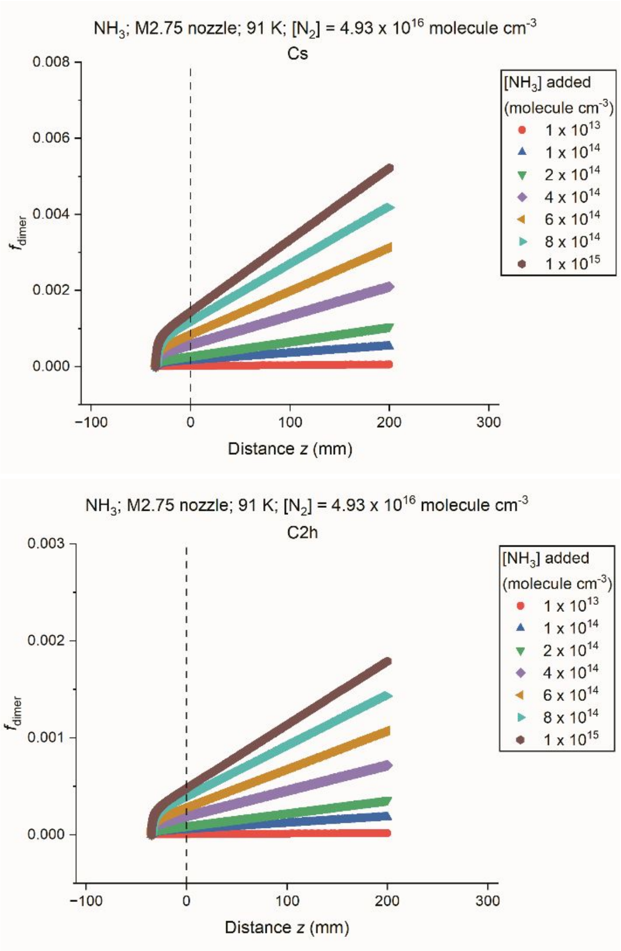

Figure S16. Calculated fraction of NH_3 dimers f_{dimer} along the gas flow as a function of distance z using the (top) "Cs" and (bottom) "C2h" models for the M2.75 nozzle in N_2 (T = 91 K, density = 4.93×10^{16} molecule cm^{-3} in the uniform flow). The values in the legend correspond to the added concentration of NH_3 (in molecule cm^{-3}) in experiments. The vertical dashed line at z = 0 indicates the nozzle exit.

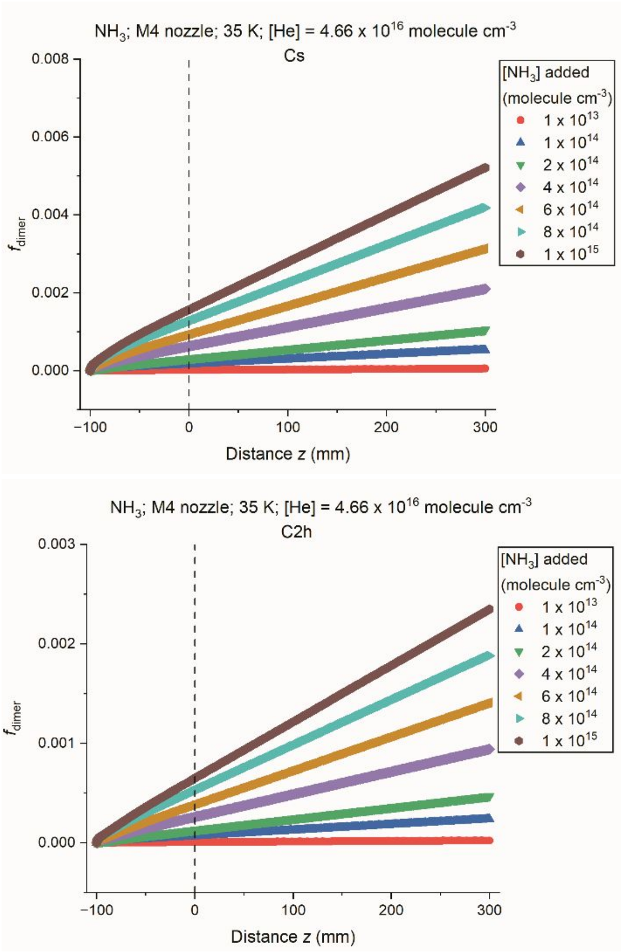

Figure S17. Calculated fraction of NH_3 dimers f_{dimer} along the gas flow as a function of distance z using the (top) "Cs" and (bottom) "C2h" models for the M4 nozzle in He (T = 35 K, density = 4.66 × 10^{16} molecule cm^{-3} in the uniform flow). The values in the legend correspond to the added concentration of NH_3 (in molecule cm^{-3}) in experiments. The vertical dashed line at z = 0 indicates the nozzle exit.

S12. $f_{monomer}$ versus distance z for various added NH_3 concentrations using different calculation models

Table S11. Decrease in $f_{monomer}$ within the nozzle and the distances z (mm) travelled in the uniform flow region required to generate the same decrease in $f_{monomer}$ for the M2.75 nozzle in N_2 and the M4 nozzle in He using the "Cs+1hindered" model.

M2.75 nozzle (L = 35 mm, T = 91 K, $[N_2]$ = 4.93 × 10^{16} molecule cm^{-3})		
$[NH_3]$ added (molecule cm^{-3})	Decrease in $f_{monomer}$ (%) within the nozzle	Further distance z (mm) travelled in the uniform flow to generate the same decrease in $f_{monomer}$ as within the nozzle
10^{13}	0.007	59
10^{14}	0.071	59
10^{15}	0.703	60
10^{16}	6.813	71
M4 nozzle (L = 100 mm, T = 35 K, [He] = 4.66 × 10^{16} molecule cm^{-3})		
$[NH_3]$ added (molecule cm^{-3})	Decrease in $f_{monomer}$ (%) within the nozzle	Further distance z (mm) travelled in the uniform flow to generate the same decrease in $f_{monomer}$ as within the nozzle
10^{13}	0.012	112
10^{14}	0.118	112
10^{15}	1.177	115
10^{16}	11.161	153

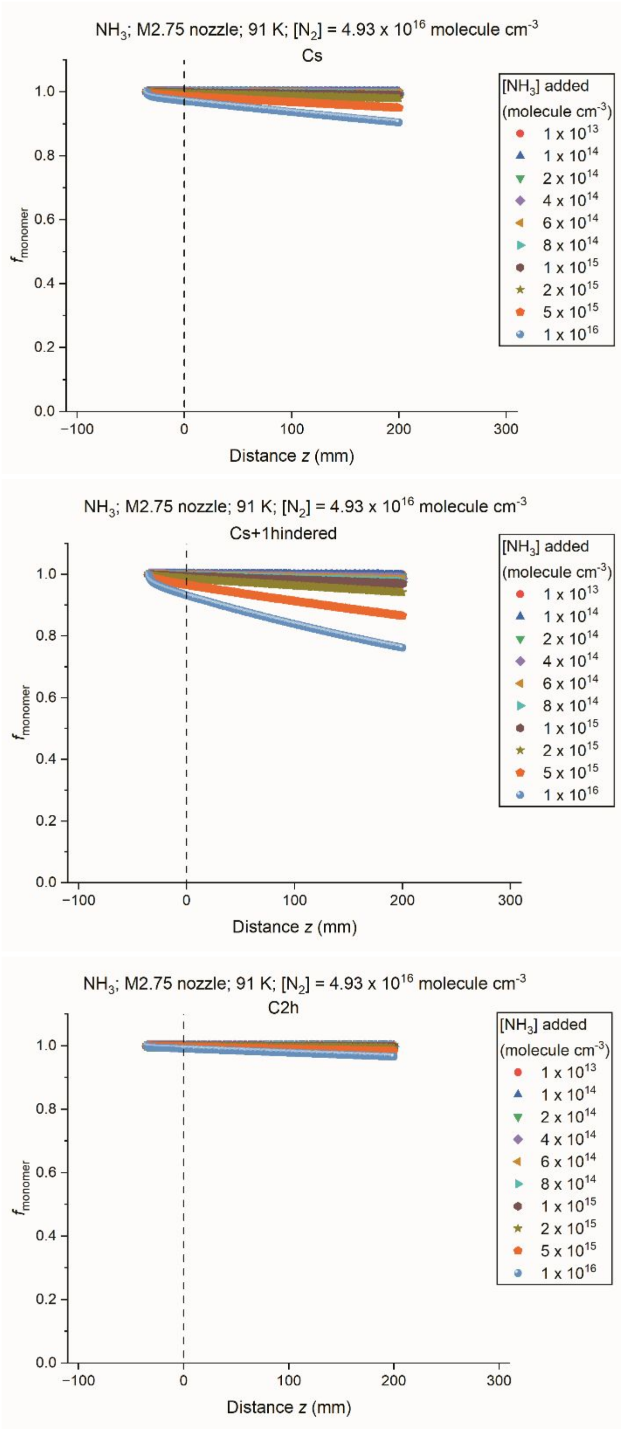

Figure S18. Calculated fraction of NH_3 monomers f_{monomer} along the gas flow as a function of distance z using different calculation models (from top to bottom: “Cs”, “Cs+1hindered”, “C2h”) for the M2.75 nozzle in N_2 (T = 91 K, density = 4.93×10^{16} molecule cm^{-3} in the uniform flow). The values in the legend correspond to the added concentration of NH_3 (in molecule cm^{-3}) in experiments. The vertical dashed line at $z = 0$ indicates the nozzle exit.

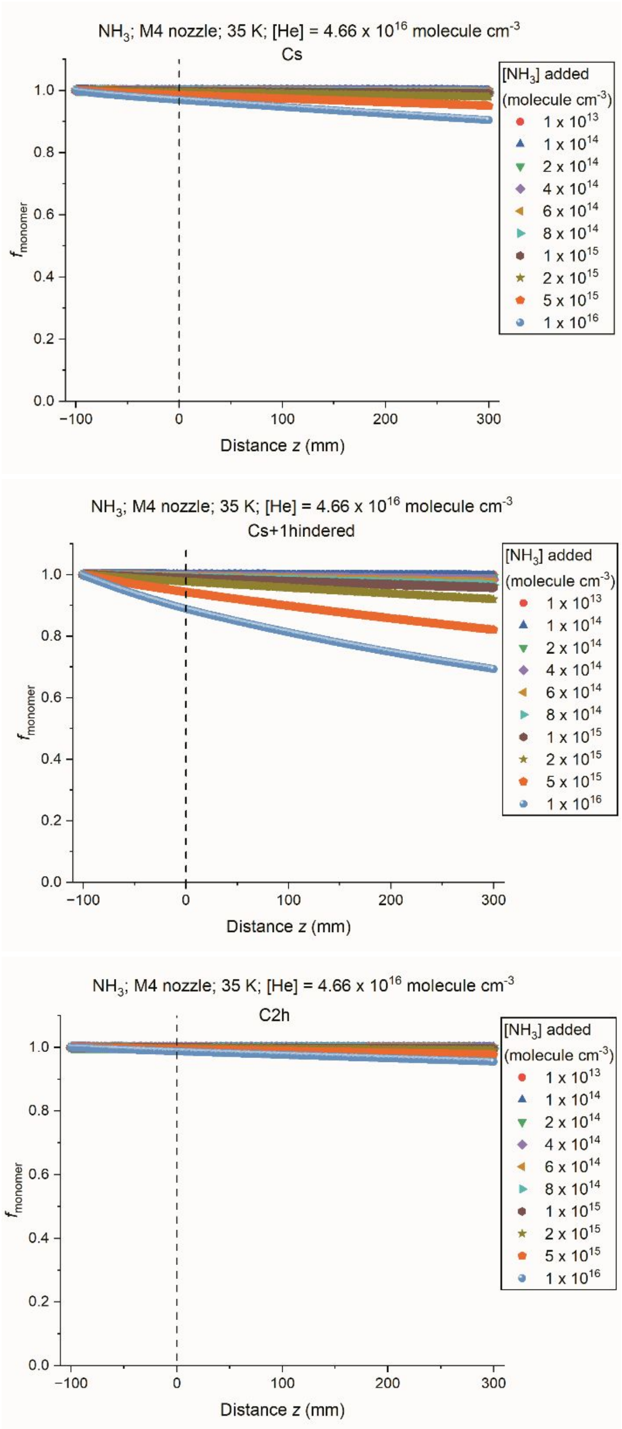

Figure S19. Calculated fraction of NH_3 monomers f_{monomer} along the gas flow as a function of distance z using different calculation models (from top to bottom: “Cs”, “Cs+1hindered”, “C2h”) for the M4 nozzle in He ($T = 35$ K, density = 4.66×10^{16} molecule cm^{-3} in the uniform flow). The values in the legend correspond to the added concentration of NH_3 (in molecule cm^{-3}) in experiments. The vertical dashed line at $z = 0$ indicates the nozzle exit.

S13. Comparison of measured and calculated f_{monomer} as a function of added NH_3 concentrations using different calculation models.

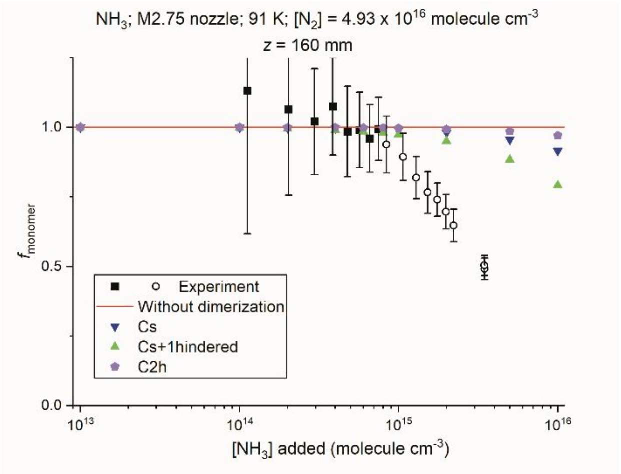

Figure S20. Fraction of NH_3 monomers as a function of the concentration of NH_3 added in experiments for the M2.75 nozzle in N_2 ($T = 91$ K, density = 4.93×10^{16} molecule cm^{-3} in the uniform flow) at $z = 160$ mm. The $f_{\text{monomer}} = 1$ red line marks the case of no dimerization. Experimental measurements, which are derived from the same data shown in Figure 1 of the main paper, are shown with closed black symbols for those that can be fitted to the red straight line (Figure 1) whilst the open symbols correspond to data at high added [NH_3] where deviation from linearity is seen (Figure 1). Calculated values from various models are shown with coloured points (blue: "Cs", green: "Cs+1hindered", purple: "C2h").

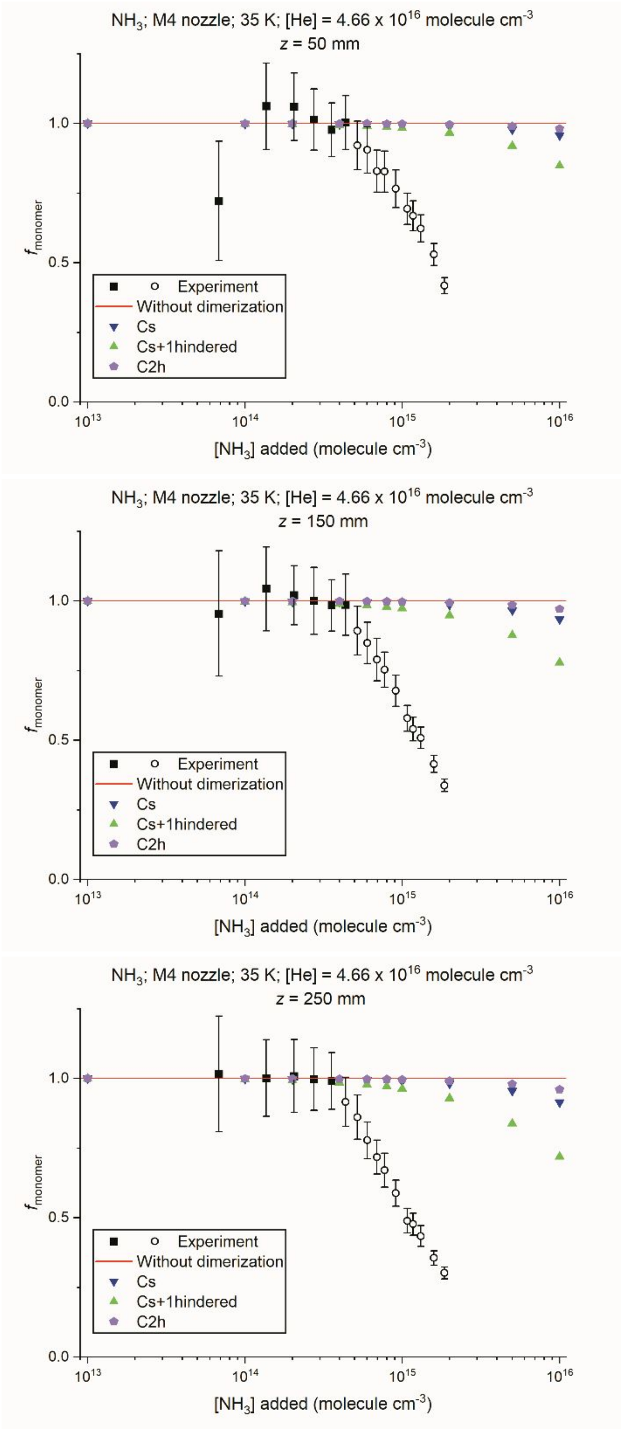

Figure S21. Fraction of NH_3 monomers as a function of the concentration of NH_3 added in experiments for the M4 nozzle in He (T = 35 K, density = 4.66×10^{16} molecule cm^{-3} in the uniform flow) (from top to bottom) at z = 50 mm, 150 mm and 250 mm. The $f_{monomer} = 1$ red line marks the case of no dimerization. Experimental measurements, which are derived from the same data shown in Figure S1, are shown with closed black symbols for those that can be fitted to the red straight line (Figure S1) whilst the open symbols correspond to data at high added [NH_3] where deviation from linearity can be seen (Figure S1). Calculated values from various models are shown with coloured points (blue: "Cs", green: "Cs+1hindered", purple: "C2h").

S14. f_{dimer} and f_{monomer} versus distance z calculated using an artificially increased k_{dimer}

While the "Cs+1hindered" model gives the largest value of k_{dimer}, it is still insufficient to match with the curvature observed for the monomer concentration in experiments. Values of k_{dimer} from the "Cs+1hindered" model need to be multiplied by a factor of 5 – 10 for the M2.75 nozzle in N_2, and a factor of 10 – 35 for the M4 nozzle in He in order to match experiment. The resulting f_{dimer} and f_{monomer} as a function of distance z with the artificially increased k_{dimer} are shown in Figures S22 and S23.

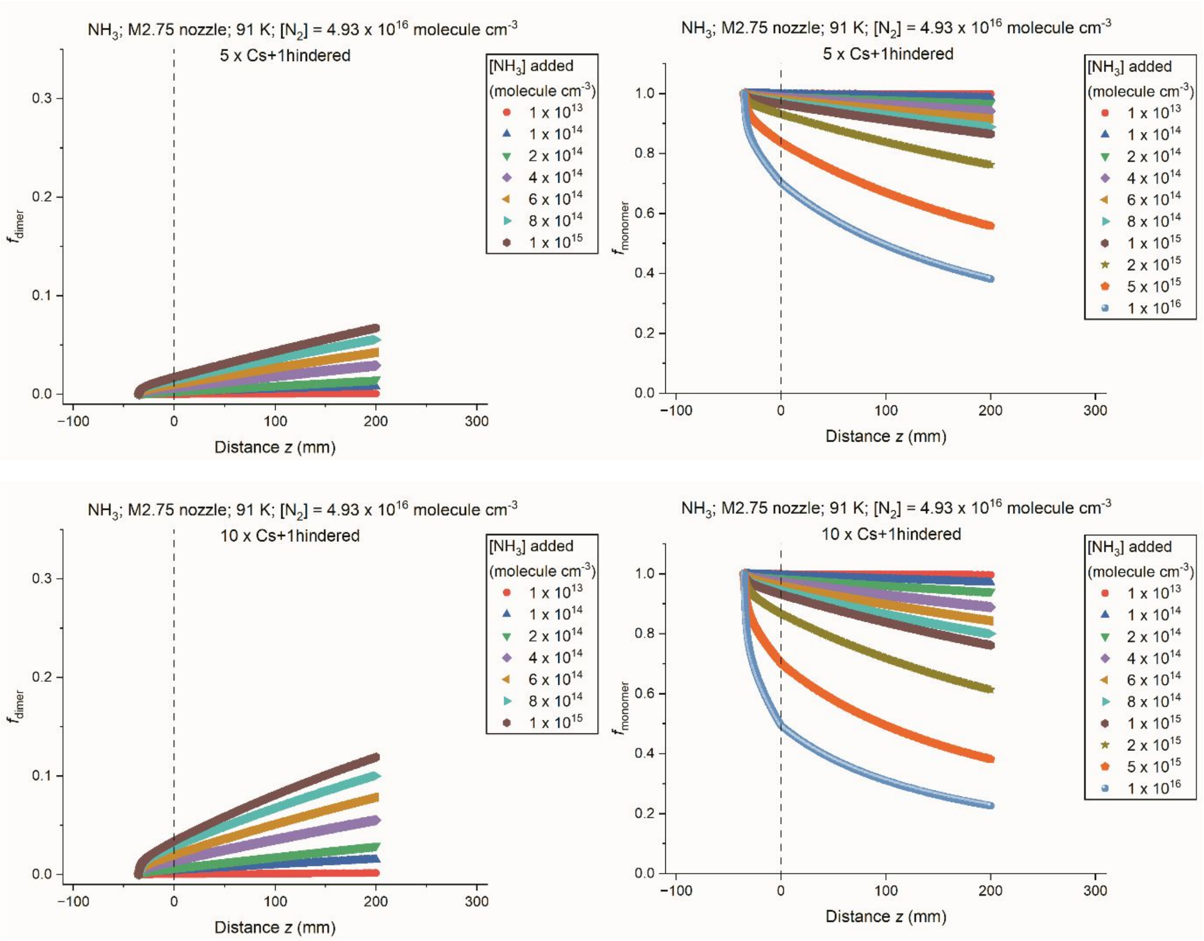

Figure S22. (Left) Fraction of NH_3 dimers f_{dimer} and (right) fraction of NH_3 monomers f_{monomer} along the gas flow as a function of distance z with the value of k_{dimer} artificially increased by a factor of (top) 5 times and (bottom) 10 times of that obtained from the "Cs+1hindered" model for the M2.75 nozzle in N_2 ($T = 91$ K, density = 4.93×10^{16} molecule cm^{-3} in the uniform flow). The values in the legend correspond to the added concentration of NH_3 (in molecule cm^{-3}) in experiments. The vertical dashed line at $z = 0$ indicates the nozzle exit.

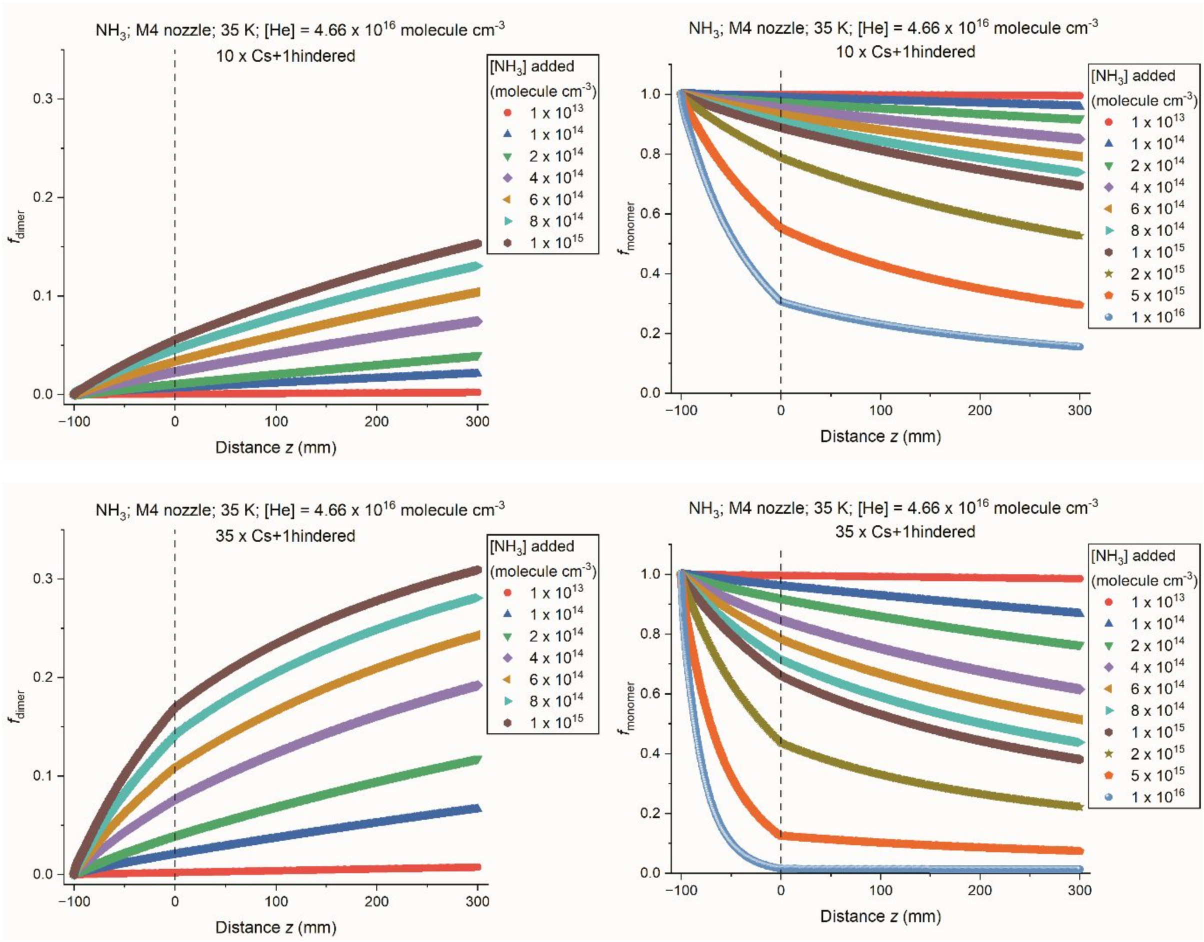

Figure S23. (Left) Fraction of NH_3 dimers f_{dimer} and (right) fraction of NH_3 monomers f_{monomer} along the gas flow as a function of distance z with the value of k_{dimer} artificially increased by a factor of (top) 10 times and (bottom) 35 times of that obtained from the "Cs+1hindered" model for the M4 nozzle in He (T = 35 K, density = 4.66×10^{16} molecule cm^{-3} in the uniform flow). The values in the legend correspond to the added concentration of NH_3 (in molecule cm^{-3}) in experiments. The vertical dashed line at z = 0 indicates the nozzle exit.

References:

(1) Frisch, M. J.; Trucks, G. W.; Schlegel, H. B.; Scuseria, G. E.; Robb, M. A.; Cheeseman, J. R.; Scalmani, G.; Barone, V.; Mennucci, B.; Petersson, G. A.; et al. Gaussian 09 Revision D.01. Gaussian, Inc.: Wallingford CT, 2013.

(2) Stoecklin, T.; Dateo, C. E.; Clary, D. C. Rate constant calculations on fast diatom–diatom reactions. *Journal of the Chemical Society, Faraday Transactions* **1991**, *87* (11), 1667-1679, 10.1039/FT9918701667. DOI: 10.1039/FT9918701667.

(3) Hirschfelder, J. O.; Curtiss, C. F.; Bird, R. B. *Molecular theory of gases and liquids*; Wiley, 1954.

(4) Smith, I. W. M. *Kinetics and dynamics of elementary gas reactions*; Butterworths, 1980.

(5) West, N. A.; Millar, T. J.; Van de Sande, M.; Rutter, E.; Blitz, M. A.; Decin, L.; Heard, D. E. Measurements of Low Temperature Rate Coefficients for the Reaction of CH with CH_2O

and Application to Dark Cloud and AGB Stellar Wind Models. *The Astrophysical Journal* **2019**, *885* (2), 134. DOI: 10.3847/1538-4357/ab480e.

(6) Troe, J. Theory of thermal unimolecular reactions at low pressures. II. Strong collision rate constants. Applications. *The Journal of Chemical Physics* **1977**, *66* (11), 4758-4775. DOI: 10.1063/1.433838 (acccessed 2022/03/23).

(7) Gilbert, R. G.; Smith, S. C. *Theory of unimolecular and recombination reactions*; Blackwell Scientific, 1990.

(8) Troe, J. Theory of thermal unimolecular reactions at low pressures. I. Solutions of the master equation. *The Journal of Chemical Physics* **1977**, *66* (11), 4745-4757. DOI: 10.1063/1.433837 (acccessed 2020/06/12).

(9) Antiñolo, M.; Agúndez, M.; Jiménez, E.; Ballesteros, B.; Canosa, A.; Dib, G. E.; Albaladejo, J.; Cernicharo, J. Reactivity of OH and CH_3OH between 22 and 64 K: modeling the gas phase production of CH_3O in Barnard 1b. *The Astrophysical Journal* **2016**, *823* (1), 25. DOI: 10.3847/0004-637x/823/1/25.

(10) Driver, L.; Douglas, K. M.; Lucas, D.; Guillaume, T.; Lehman, J. H.; Kapur, N.; Heard, D. E.; de Boer, G. Developing a Predictive Model for Low Temperature Laval Nozzles with Application to the CRESU Method. *Physics of Fluids* **2024**.
